# ImiRP: a computational approach to microRNA target site mutation

**DOI:** 10.1186/s12859-016-1057-y

**Published:** 2016-04-27

**Authors:** Bridget C. Ryan, Torben S. Werner, Perry L. Howard, Robert L. Chow

**Affiliations:** Department of Biology, University of Victoria, Victoria, BC V8W 3N5 Canada; Department of Biochemistry and Microbiology, University of Victoria, Victoria, BC V8W 3N5 Canada

**Keywords:** MicroRNA, Mutagenesis, Software, Automation, Target Site, Seed Sequence

## Abstract

**Background:**

MicroRNAs (miRNAs) are small ~22 nucleotide non-coding RNAs that function as post-transcriptional regulators of messenger RNA (mRNA) through base-pairing to 6–8 nucleotide long target sites, usually located within the mRNA 3’ untranslated region. A common approach to validate and probe microRNA-mRNA interactions is to mutate predicted target sites within the mRNA and determine whether it affects miRNA-mediated activity. The introduction of miRNA target site mutations, however, is potentially problematic as it may generate new, “illegitimate sites” target sites for other miRNAs, which may affect the experimental outcome. While it is possible to manually generate and check single miRNA target site mutations, this process can be time consuming, and becomes particularly onerous and error prone when multiple sites are to be mutated simultaneously. We have developed a modular Java-based system called ImiRP (Illegitimate miRNA Predictor) to solve this problem and to facilitate miRNA target site mutagenesis.

**Results:**

The ImiRP interface allows users to input a sequence of interest, specify the locations of multiple predicted target sites to mutate, and set parameters such as species, mutation strategy, and disallowed illegitimate target site types. As mutant sequences are generated, ImiRP utilizes the miRBase high confidence miRNA dataset to identify illegitimate target sites in each mutant sequence by comparing target site predictions between input and mutant sequences. ImiRP then assembles a final mutant sequence in which all specified target sites have been mutated.

**Conclusions:**

ImiRP is a mutation generator program that enables selective disruption of specified miRNA target sites while ensuring predicted target sites for other miRNAs are not inadvertently created. ImiRP supports mutagenesis of single and multiple miRNA target sites within a given sequence, including sites that overlap. This software will be particularly useful for studies looking at microRNA cooperativity, where mutagenesis of multiple microRNA target sites may be desired. The software is available at imirp.org and is available open source for download through GitHub (https://github.com/imirp).

**Electronic supplementary material:**

The online version of this article (doi:10.1186/s12859-016-1057-y) contains supplementary material, which is available to authorized users.

## Background

MicroRNAs (miRNAs) are 21-25 nucleotide non-coding RNAs that provide rapid repression of target gene expression. This repression is initiated through miRNA base pairing to complementary target sites usually located within messenger RNA (mRNA) 3’ untranslated region (3’UTR) [1, 2]. Computational and experimental evidence suggest that the 5’ end of the miRNA, the miRNA “seed”, is most important for mediating miRNA-target interactions [3–7]. The miRNA seed recognizes a complementary target site in the mRNA, which can range from six to eight nucleotides in length [2]. MiRNAs act as sequence-specific guides that recruit protein silencing complexes and destabilize the mRNA and/or interfere with translation [8], and thereby provide a mechanism for either silencing or fine-tuning the translation of target mRNAs [9, 10].

Short RNA deep-sequencing data has identified over 15000 miRNA gene loci and over 17000 mature miRNA sequences in 142 species. Specifically, over 2500 and 1900 distinct mature miRNA sequences have been identified in human and mouse, respectively [11]. Computational approaches that take into account evolutionary conservation of predicted target sites suggest that over 60 % of human protein-coding genes are targeted by miRNAs [12], while other computational methods using pattern-based approaches for predicting miRNA-target heteroduplexes estimate that over 90 % of mammalian gene transcripts are directly regulated by miRNAs [13]. In addition, a growing body of evidence reveals that miRNAs play important roles during animal development [14] and almost every cellular process investigated has been shown to involve participation of miRNA regulation [10]. Due to their ability to impose rapid and tight control of gene expression, miRNAs represent an important regulatory mechanism for development and disease.

Many computational tools have been developed for studying miRNAs. For example, several of programs are available to address challenges associated with miRNA discovery [15, 16] and numerous applications enable identification of miRNA target sites in mRNAs [3, 12, 13, 17–26]. MicroRNA target site prediction programs make use of a number of different parameters in their predictions, such as extent of complementarity to the miRNA 5’ end, hybridization energy of the mRNA-miRNA heteroduplex, evolutionary conservation of predicted target sites, mRNA secondary structure, and local 3’UTR context. Additionally, other software tools are available for predicting the impact of 3’UTR SNPs on miRNA binding [26], for assessing the impact of mutations in miRNA genes [27], and for comparing the predicted set of target genes for two different miRNAs [27].

**Fig. 1:**
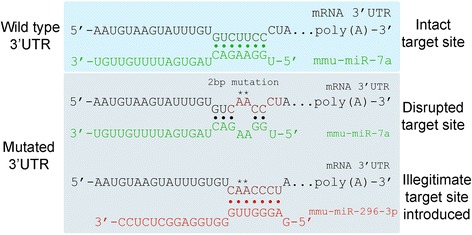
The problem associated with miRNA target site mutagenesis. A wild type 3’UTR sequence contains a predicted 7mer-m8 target site for miR-7 (*green*). Mutation disrupts the interaction between miR-7 and its predicted target site, but creates an illegitimate 7mer-m8 target site for miR-296-3p in the process (*red*)

Currently, no software is available for generating mutations to disrupt predicted miRNA target sites in a given mRNA sequence. For example, although the program “mrSNP”, was designed to take known SNPs for a given 3’UTR as input and assesses whether these SNPs could affect miRNA binding on the other hand, it is not a mutation generator program and does not have the capacity to generate random mutations. Generating such mutations, however, is an important approach for examining miRNA-mRNA interactions experimentally. Mutating a predicted miRNA target site is commonly used in reporter-based approach experiments to determine whether a predicted target site can, in fact, be regulated by a candidate miRNA. In addition, mutations that disrupt one or more miRNA target sites in a given 3’ UTR can be used to examine the biological relevance of the target site(s) in question. One problem that can arise when a target site is mutated is that the mutation itself can create a new or “illegitimate” microRNA target site (Fig. 1). If the miRNA that targets the illegitimate site is present in the cell being used in the experiment, it could confound the data. Therefore, care must be taken when devising a mutation strategy to check that target site mutations do not inadvertently create new miRNA target sites.

Our goal was to develop a modular, open source application that could be run through a web interface or downloaded and adapted to specialized projects. We have created a Java-based system called ImiRP (Illegitimate miRNA Predictor) to generate miRNA target site mutations in any sequence of interest and address the problem of illegitimate miRNA target site creation. Users input a DNA or RNA sequence and specify the location of predicted miRNA target sites to be mutated. ImiRP then automates the processes of mutation generation, illegitimate miRNA target site identification, and synthesis of a final mutant sequence lacking new predicted miRNA target sites. ImiRP is a particularly useful tool when investigating regulation of a single mRNA 3’ UTR by multiple miRNAs, where situations such as target site overlap makes mutagenesis design more challenging to perform manually.

## Implementation

### Input user interface

**Fig. 2:**
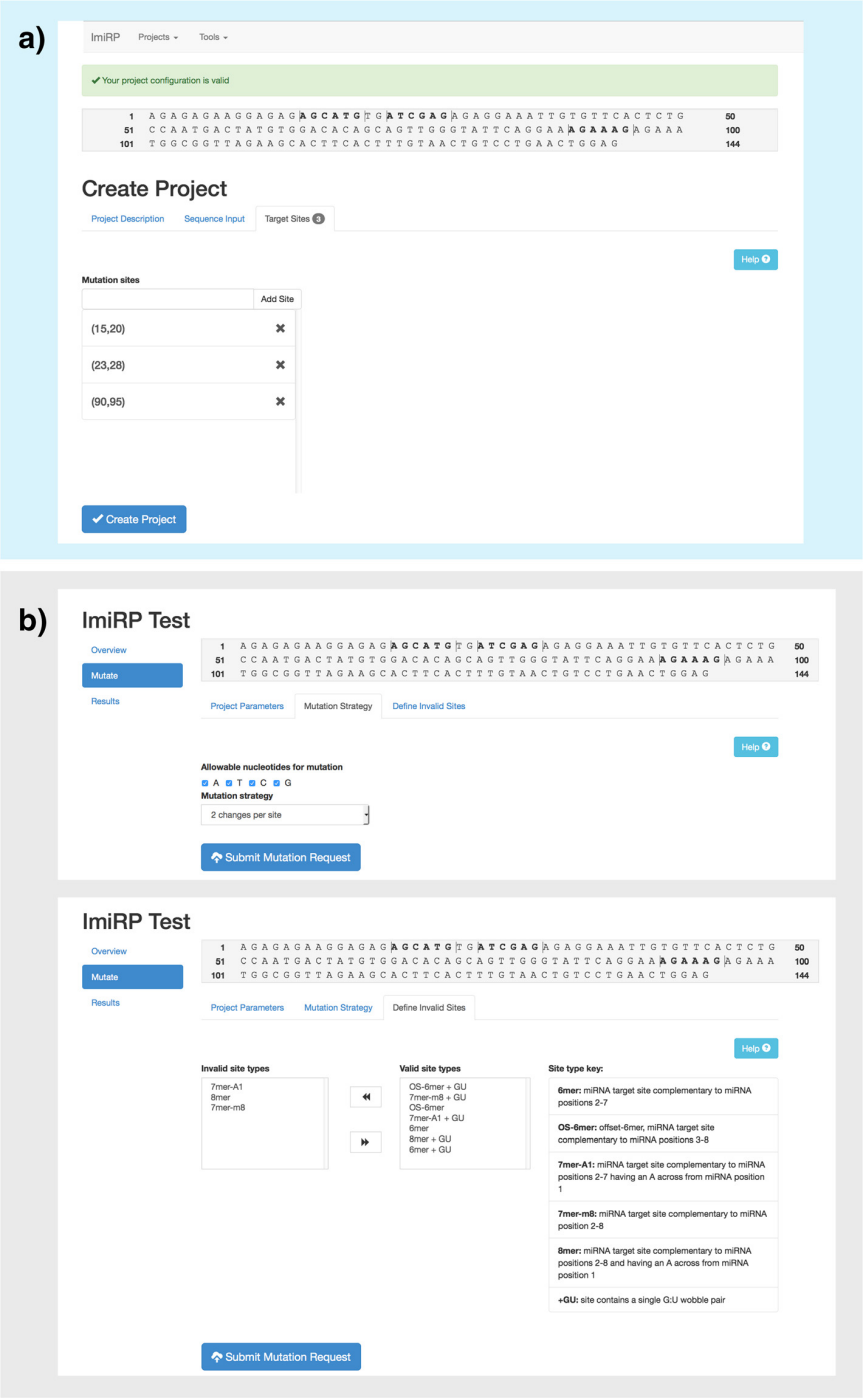
ImiRP input user interface. **a** The user is first directed to create a project and specify project parameters. The project can be named, and in this example we have named the project “ImiRP Test”. A DNA or RNA sequence must be input along with information about the species of interest. The input sequence will be displayed for viewing. Finally, at least one “mutation site”, the region into which mutations will be introduced, is specified by typing the sequence position complementary to miRNA position 7 into the textbox. All selected mutation sites, complementary to miRNA positions 2-7, appear *bolded* in the displayed original input sequence for inspection. In this example, a segment of the mouse *Pax6* 3’UTR and three hypothetical predicted target sites have been used as input. **b** Upon creation of a project, the user is asked to specify mutation parameters. In the “Mutation Strategy” tab, nucleotides to use for mutation and number of nucleotide changes per specified mutation site can be selected. In the “Define Invalid Sites” tab, users can specify the types of newly created miRNA target sites they do not want present in their final mutant sequence. In this case, any mutant sequences provided as output will contain two adjacent nucleotide changes per specified target site using all four nucleotides for mutation, and no mutant sequences containing newly created 8mer, 7mer-m8, or 7mer-A1 predicted target sites will be provided as output

The ImiRP user interface showing examples of input and output are shown in Figs. 2 and 6. A detailed set of instructions, “*How to use ImiRP*” is appended in the Additional file 1 section as well as on the imirp.org website. After creating a new project, the user enters a DNA or mRNA sequence of interest (i.e. 3’UTR) and specifies the organism of interest. All animal species with mature miRNA sequences available through miRBase version 21 (mirbase.org) are available for selection [11]. Users then define the position(s) of predicted miRNA target sites(s) to be mutated by selecting the nucleotide position of the input sequence that is complementary to position 7 of the targeting miRNA (Fig. 2a). ImiRP will then highlight in bold lettering, a stretch of 6 nucleotides starting from the nucleotide position that was entered, defining the mRNA region, complementary to miRNA positions 2-7, to be mutated (Fig. 2a). Thus, for 7mer-m8 target sites, in which base pair complementarity occurs at miRNA positions 2-8, the input nucleotide position that is complementary to miRNA position 7 is entered in the “Mutation Site” input window.

In designing ImiRP, we took into consideration the fact that miRNA target sites vary in size and type (e.g. 6mer, 7mer-m8, 7mer-A1 or 8mer) [2, 12]. We chose this design strategy for defining mutation sites two reasons: 1) To keep ImiRP simple to use and to limit computational demands of the software. MicroRNA seed site positions 2-7 are used for mRNA target recognition in all major target site types [2, 12]. 2) Disrupting base pairing between miRNA positions 2-7 and the mRNA target site by mutating multiple nucleotides in the target site has been shown to interfere with repression [7, 28–31].

Upon defining the project parameters, the ImiRP user interface allows researchers to specify mutation parameters (Fig. 2b). The user can select the types of illegitimate miRNA target sites, termed “invalid sites”, that they wish to exclude from their mutant sequence. ImiRP can identify five classes of miRNA target sites: 6mer, 7mer-m8, 7mer-A1, 8mer, and offset 6mer (OS-6mer) [2, 12]. 6mer seed sites are complementary to positions 2-7 of the miRNA, 7mer-A1 sites are complementary to positions 2-7 and have an A across from miRNA position 1, and 8mer sites are 7mer-m8 sites with an A across from position 1 [12]. OS-6mer seed sites are complementary to positions 3-8 of the miRNA [12]. ImiRP also recognizes predicted target sites containing a single G:U base pair. By manually selecting and using arrow buttons in the user interface, invalid site types can be designated (Fig. 2b). For instance, illegitimate perfect 6-8mer target sites can be specified as invalid by moving them into the select list entitled “Invalid Site Types”. In this case, the result would be a final mutant sequence lacking newly created perfect 6-8mer sites, but permitting newly created 6-8mer sites containing a single G:U wobble base pair. The interface also enables the user to specify a desired mutation strategy to be executed by selecting which nucleotide bases to use for mutagenesis from a checklist. Selecting only G will instruct ImiRP to generate mutant sequences by making nucleotide changes within specified target sites to G only. Using a drop down menu, the number of nucleotide changes to introduce per specified miRNA target site, from two to six changes, can be designated. For example, selecting “2 changes per site” will ensure that two adjacent nucleotide changes are introduced into each selected miRNA target site.

### ImiRP workflow

**Fig. 3:**
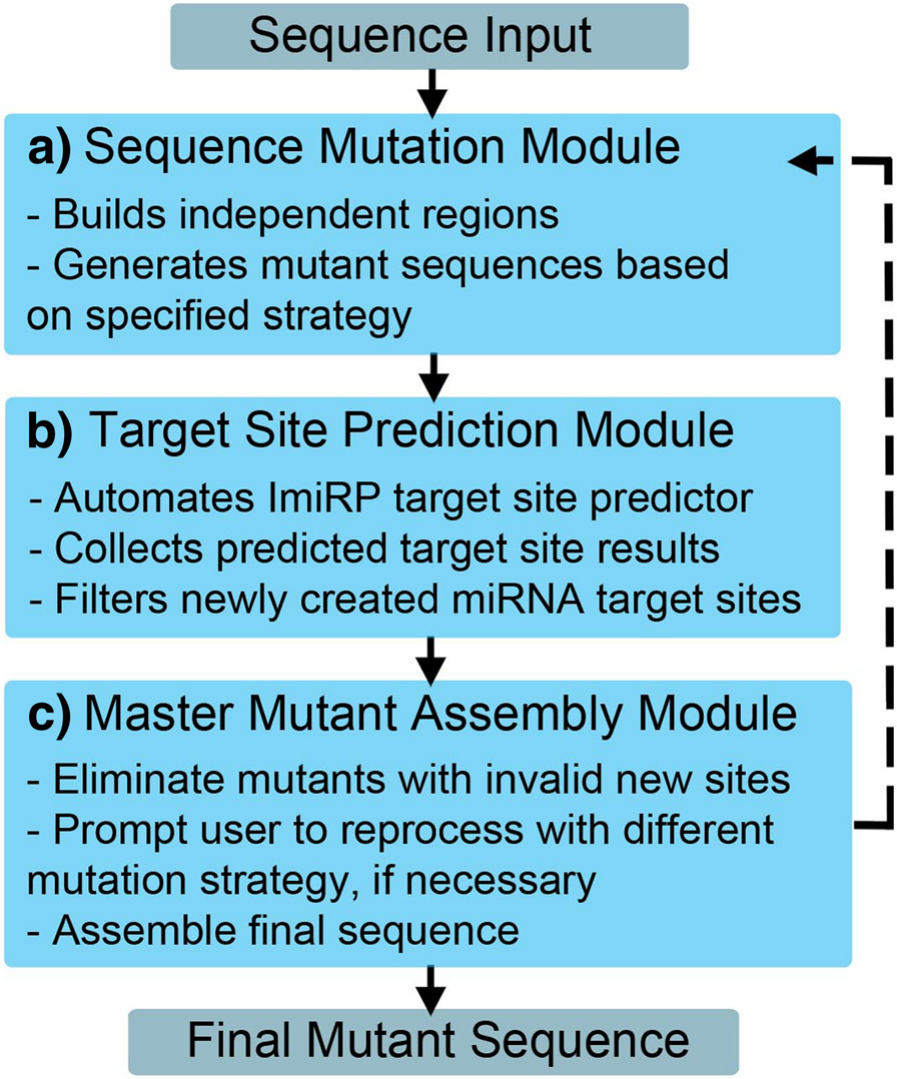
ImiRP Workflow. ImiRP can be divided into three major modules. The Sequence Mutation module **a** feeds mutated sequences into the Target Site Prediction module **b**, the results of which are supplied to the Master Mutant Assembly module **c** to optimize and output a final mutant sequence

**Fig. 4:**
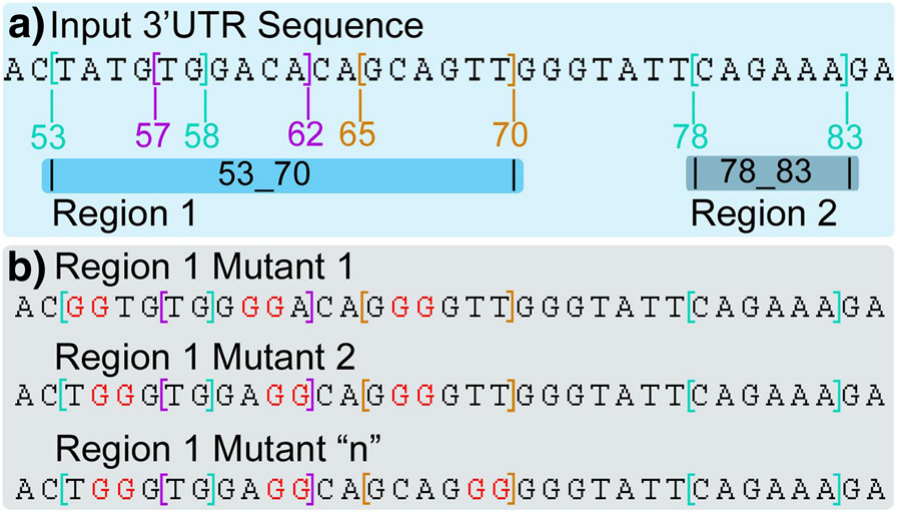
The Sequence Mutation module. **a** The input sequence is divided into “mutationally independent” regions. User-specified target sites spaced less than 7 nucleotides apart are grouped into a single independent region and are annotated based on the positions of the first and last nucleotides of sites within the given region. **b** To reduce computational overhead, sites within a given independent region are mutated as a unit, while sites in other regions are unchanged
ii) *Target site prediction module*
  Mutant 3’UTR sequences generated by the Sequence Mutation module are sent to the Target Site Prediction module (Fig. 3b). This module is responsible for identifying predicted miRNA target sites present in each mutant sequence that are absent from the original input sequence. The custom ImiRP miRNA prediction component uses pattern recognition to identify regions of each mutant 3’UTR sequence that are complementary to the 5’ seed regions of known mature miRNAs. High confidence FASTA-format miRNA sequences were obtained from the most recent release of miRBase, version 21 [11]. MiRBase defines a high confidence miRNA sequence as having at least ten reads mapping to each arm of the pre-miRNA, or having at least five reads mapping to each arm and at least 100 reads in total [11].
  Following miRNA target site identification, the prediction module compares the input sequence target site predictions to predictions for each mutant sequence. Information about predicted sites present in each mutant sequence that are absent from the input sequence (i.e. the illegitimate target sites) are stored in a database along with the respective mutant sequences (Fig. 5). Information in the database is accessed by the Master Mutant Assembly module (Fig. 3c) for synthesis of a final mutant sequence.

Conceptually, ImiRP can be subdivided into three modules (Fig. 3):

i) *Sequence mutation module*
  When ImiRP is run, information about the input sequence, defined regions to mutate, and desired mutation strategy are sent to the Sequence Mutation module (Fig. 3a). This module first divides the input sequence into “mutationally independent” regions based on the spacing between miRNA target sites to be mutated. Since the largest recognized miRNA seed site is eight nucleotides [2], two predicted target sites spaced at least seven nucleotides apart can be mutated independent of one another without generating an illegitimate target site that spans both mutations. Simultaneously mutating two predicted target sites spaced less than seven nucleotides apart could generate a new target site for a different miRNA containing mutated nucleotides from each of the original target sites. Consequently, predicted target sites spaced less than seven nucleotides apart are grouped into a single independent region, and each independent region is annotated based on the positions of the first and last nucleotides of sites within the region relative to start of the input sequence (Fig. 4a). All specified sites within a given independent region are mutated as a unit, while sites within other regions are left unchanged. This process is repeated for each independent region (Fig. 4b).

**Fig. 5:**
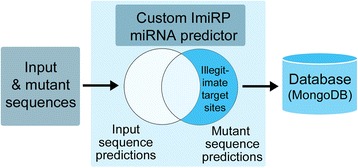
The target site prediction module. Predicted miRNA target sites within input and mutant sequences are identified using a custom component that identifies predicted target sites based on complementarity to the miRNA 5’ end. FASTA-format miRNA sequence data was collected from miRBase version 21 [11]. Information about predicted sites present in each mutant sequence that are absent from the input sequence are stored in a database for analysis
iii) *Master mutant assembly module*
  The Master Mutant Assembly module first eliminates all mutant sequences containing illegitimate miRNA target sites that were specified as invalid through the user interface. This process is repeated for each independent region. As valid mutations for each region are generated, they are displayed in an output user interface (Fig. 6). The output interface displays up to five valid mutants for each independent region, indicating the location of specified six nucleotide “mutation sites” and mutated nucleotides. Here, the user can select a single desired mutant for each independent region and ImiRP generates an assembled mutant sequence in which all specified miRNA target sites have been mutated without creating any illegitimate predicted target sites of the types specified. A zip folder containing four files is made available for download through the output user interface. A project information text file contains the assembled mutant DNA sequence, and three CSV files contain information about: i) miRNA target site predictions for the input sequence, ii) miRNA target site predictions for the assembled mutant sequence, and iii) new predicted miRNA target sites present in the mutant sequence. If any regions fail to generate valid results, the user will be alerted to reprocess those regions using a different mutation strategy. Accumulated results from multiple mutation runs are displayed in the results tab of the output user interface. In the future, we plan to annotate each valid mutant sequence displayed in the results with a “mutation run identifier” so that it is clear which mutation parameters were used to generate each sequence.

**Fig. 6:**
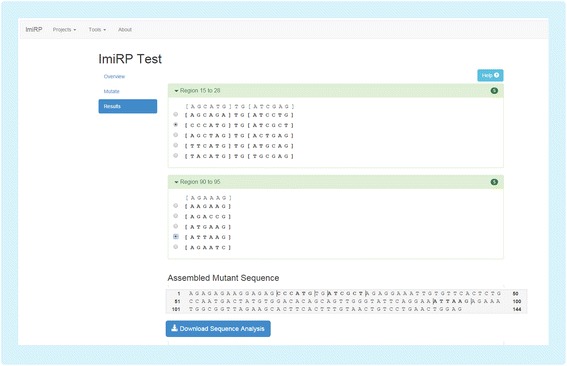
ImiRP output user interface. Input mutation sites spaced less than seven nucleotides apart are grouped into independent regions and each region is mutated independently of other regions. The output user interface displays up to five mutant sequences for each region that satisfy the specified mutation parameters. For example, the output mutations for ImiRP Test (Fig. 2) will not have generated any new 8mer, 7mer-m8, or 7mer-A1 predicted miRNA target sites. The original input sequence for each region is displayed above the mutants for comparison, and brackets denote the bounds of each specified mutation site. The user must select one desired mutant for each region, and the changes are displayed as an assembled mutant sequence. Once the selection process is complete, the user may download a folder containing information about their assembled mutant sequence, and predicted miRNA target sites in the input and mutant sequences

### Project organization

ImiRP’s code is organized into two separate Scala Build Tool (SBT) projects: ImiRP Core and ImiRP Web. ImiRP Core contains the data model and the primary business logic but has no presentation layer. Core functionality is exposed through Java services and Akka actors, and must be programmatically interacted with. ImiRP Web depends on ImiRP Core and exposes the functionality of ImiRP Core to an end user via a Web interface. This multi-project organization scheme was chosen to keep the presentation logic separate from the core business logic, thus allowing ImiRP to support alternative core or presentation implementations in the future. Additionally, this organizational approach allows ImiRP to be run headless (without a presentation layer) or to be integrated with other projects or systems as a library.

### Mutant sequence generation algorithm

ImiRP’s mutation generation algorithm is essentially a random nucleotide sequence permutation generator. As input, it accepts a DNA or RNA nucleotide sequence, a list of sites, a set of allowable nucleotide types for mutagenesis, number of allowable nucleotide changes per site, a mutation region, and a callback. The algorithm iterates over each site in the given independent mutation region to apply nucleotide changes to each site. To accomplish this, the algorithm selects a random nucleotide from the list of allowable nucleotides and replaces the nucleotide in the input sequence at the start of the site. This process is repeated for subsequent nucleotides contained within the given site. A mutant sequence is produced once all sites have been processed in this way. Due to the simplicity of this algorithm, some mutant sequences will not satisfy all specified mutation criteria. As such, all mutant sequences must be passed through a filter that either accepts or rejects the mutant based on whether it satisfies all criteria. Finally, if a mutant sequence is accepted by the filter, the callback is activated and the mutant sequence is passed on for further processing (see Additional file 1: Figure S2 for pseudocode).

### MicroRNA target site prediction algorithm

The target site predictor uses a pattern matching algorithm to detect miRNA seed matches to an input sequence. To detect seed matches, the algorithm iterates over a list of miRNA sequences, from the miRBase high confidence database, and compares the first eight nucleotides (the 5’ end) of each miRNA entry for complementarity to the input sequence. An entry is compared to a sequence by scanning across the sequence using an eight nucleotide sliding window. Before each advancement of the window, a hierarchy of conditions are checked to determine whether a seed match is present. If a seed match is found, the result is stored, the window is advanced, and the process repeats until the end of the sequence is reached.

The condition hierarchy begins by comparing the first nucleotide (position 1) of the sequence window to the last nucleotide of the miRNA 5’ end (position 8). If those nucleotides are a match, the next five nucleotides (positions 2-6 of the sequence, positions 7-3 of the miRNA 5’ end) are checked for matches. If any of these nucleotides are not a match, no seed match is possible and the window will advance. If they do match, then an 8mer, 7mer-m8, or OS-6mer seed match is known to be present. The condition hierarchy is then further evaluated to check for a match between sequence position 7 and miRNA position 2. If this match is not present, then an OS-6mer seed match is identified. If this match is present, the seed match is either an 8mer if the last nucleotide of the sequence window (position 8) is an adenosine, or it is a 7mer-m8 if the last nucleotide is not adenosine. If the first nucleotide of the sequence window did not match miRNA position 8, then only a 7mer-A1 or a 6mer seed match are possible, given that sequence positions 2-7 and miRNA positions 7-2 have been found to match. The seed match is a 7mer-A1 if the last nucleotide of the sequence window is adenosine, or the seed match is a 6mer if the last nucleotide is not adenosine. Two nucleotides are considered a match if the following conditions are true: the sequence nucleotide is A and the miRNA 5’ end nucleotide is T/U or vice versa, the sequence nucleotide is C and the miRNA nucleotide is G or vice versa. If the user allowed G:U pairs and if a G:U pair has not already been found, two nucleotides will be considered a match if the sequence nucleotide is T/U and the miRNA nucleotide is G or vice versa (see Additional file 1: Figure S3 for pseudocode).

### System architecture

ImiRP was developed in the Java programming language. Java was chosen for its extensive catalogue of third party libraries and for its cross-platform support.

ImiRP’s architecture is based on a model-view-controller (MVC) architecture consisting of a view layer for presenting information, a controller layer for applying business logic, and a model layer for storing information. In its current form, the view layer is driven by the Play Framework (Play) and AngularJS. Play accepts incoming HTTP requests and maps those requests to view templates. These view templates are partially rendered by Play and then returned as HTML and Javascript for final rendering by AngularJS in a user’s browser. The controller layer is a shared responsibility between ImiRP Web and ImiRP Core. ImiRP Web’s portion of the controller layer is responsible for mapping HTTP requests to ImiRP Core’s services and returning results from those services as rendered templates. Currently, the model layer exists entirely within ImiRP Core, but this may change in the future as ImiRP Web may require some of its own models for features that are specific to presentation.

### miRBase data import

Currently, known miRNA data is imported from the high confidence dataset (mature.fa) that is provided by miRBase version 21. Mature.fa is a text file database of FASTA-format, mature miRNA sequences. ImiRP parses this database and imports it into its own internal format for use by ImiRP’s target site predictor. To accomplish this, an import utility that could read the mature.fa dataset and import miRNAs was created. The import utility was designed to allow alternative miRNA datasets to be imported provided that they follow a similar FASTA format. This enables ImiRP to stay current as new miRNA datasets become available. Presently, an alternative miRNA dataset can only be used by altering ImiRP’s global configuration file and restarting the application. In future versions of ImiRP, we may enable use of custom miRNA datasets that could be uploaded for specific projects. This way, each individual project could specify a miRNA dataset that best meets its requirements.

## Results and discussion

In order to facilitate the generation of miRNA target site mutations in the mRNA 3’ UTR, we have designed ImiRP, a program that automates the entire process of generating mutant sequences. A key feature of ImiRP is its ability to suggest mutations that lack illegitimate miRNA target sites as such sites have the potential to complicate experiments aimed at examining miRNA-target regulation.

### Computational time optimization

The number of sequences generated by the Sequence Mutation module increases rapidly with increasing predicted target sites to mutate. Each of these mutant sequences then needs to be processed to identify any illegitimate target sites generated. As a result, one problem that needed to be addressed in the implementation of ImiRP was the large computational time required to generate a final mutant sequence when mutating many predicted miRNA target sites simultaneously. We implemented several strategies below to address this problem:

i) *Limit miRNA target site mutations to six nucleotide motifs*
  Nucleotide changes introduced into the region complementary to miRNA positions 2-7 have been found to create the most significant reduction in regulation by the targeting miRNA [28]. Additionally, by limiting the problem space for target site mutagenesis to six nucleotide regions, we were able to reduce the number of possible mutant sequences and thus the computation time. A limitation to this approach is that some examples of “seedless” miRNA target sites have been identified. These rely more heavily on binding the 3’ end of the miRNA, or contain mismatches or bulges between the miRNA seed and its target [32, 33]. However, comparative analysis of orthologous mRNAs suggests that target sites complementary to the 5’ end of the miRNA are more evolutionarily conserved than expected by chance [12]. Additionally, mRNA and protein expression analysis reveals that messages containing miRNA seed matches are preferentially regulated by miRNAs [5, 6]. As such, we decided to focus on an approach that restricts mutagenesis to the six nucleotide region complementary to miRNA position 2-7.
ii) *Introduce adjacent nucleotide changes per specified seed site*
  Even in the presence of extensive 3’ pairing, single nucleotide mismatches introduced into a miRNA target site complementary to miRNA positions 2-7, the core target site, have a large impact on target site efficacy [28]. Based on these results, we reasoned that a minimum of two nucleotide changes in this region would effectively abolish regulation by the targeting miRNA [28]. Additionally, requiring that nucleotide changes be adjacent reduces the number of possible mutants generated, improving processing speed, while also reducing the number of illegitimate target sites created by narrowing the region of sequence that is altered. For an independent region containing three non-overlapping predicted target sites, over 60 billion different mutants are possible when any combination of at least two nucleotides within a core target site are mutated to any permutation of the four nucleotide bases (Additional file 1: Figure S1-a). However, when only two adjacent nucleotide changes are permitted per site, the number of possible mutant sequences for an independent region containing three non-overlapping sites is reduced to approximately 90,000 (Additional file 1: Figure S1-b). It should be noted that through the input user interface, it is possible to select a mutation strategy that will introduce more than two nucleotide changes per specified target site.
iii) *Divide the input sequence into independent regions based on inter-site spacing*
  Since 8mer target sites are the largest recognized sites [2], predicted miRNA target sites spaced seven or more nucleotides apart can be mutated without generating a new predicted miRNA target site that spans both mutations. As such, sites spaced less than seven nucleotides apart are grouped into independent regions, and are all mutated as a unit. Mutating one region at a time, while keeping the remainder of the sequence unchanged, significantly reduces the number of permutations of redundant mutations that are generated (Additional file 1: Figure S1-c) and dramatically improves processing speed. Expanding on the above example, if two independent regions containing three non-overlapping predicted target sites are mutated together, over 8 billion mutant sequences are possible. However, if these two regions containing three sites each are mutated separately of one another, approximately 180,000 mutant sequences are possible (Additional file 1: Figure S1-d).
iv) *Custom ImiRP miRNA target site prediction component*
  Initially, we used the PITA executable in the Target Site Prediction module. PITA assigns each predicted miRNA target site a score based on the difference between the free energy of miRNA-target duplex formation and the cost of unpairing mRNA secondary structure in the region of the predicted target site [23]. Though evidence suggests that miRNA target site accessibility [23] and local target site environment [34, 35] can impact target site efficacy, some evidence also suggests that mRNA secondary structure is an insufficient predictor of target site functionality [35]. Since we are primarily concerned with creating illegitimate target sites having the potential to be functional, this feature of the PITA target site prediction component was not needed. ImiRP’s custom target site predictor uses only pattern recognition to identify five types of target seed site (8mer, 7mer-m8, 7mer-A1, 6mer, OS-6mer) [2, 12] and is therefore less computationally demanding, thus improving processing time.
  Though ImiRP is capable of identifying miRNA target sequences, it is not recommended for use as a target site prediction tool. Since it predicts miRNA seed matches based only on sequence complementarity to miRNA 5’ ends, it is an over-predicting tool and will produce many false-positive predictions. Many superior programs are available to address the problem of miRNA target identification [3, 12, 13, 17–25].
v) *Stop generating mutant sequence once five valid mutants have been identified*
  The ImiRP web application needed to be designed such that many users can run projects simultaneously. Consequently, we wanted a avoid situations where a single user could consume a large portion of the web server’s resources. The ImiRP application stops generating mutant sequences once at least five valid mutants have been identified for each independent region or once the Sequence Mutation module has effectively exhausted all mutation possibilities. This ensures that a single mutation request will not continue to process unnecessary sequences and continue to consume resources. Due to the Sequence Mutation module generating mutations faster than the Target Site Prediction module can scan them; it is possible that more than five valid mutations may be identified. Only five valid mutant sequences are displayed for each independent region in the results tab of the output user interface. If a user is interested in having all valid mutations displayed, they can download the source code and run the application on their own machine.

### Generation of mutant sequences

We made several attempts to devise an algorithm that would not generate duplicate mutant sequences or mutants that did not satisfy all criteria. In an attempt at preventing duplicate mutations, a sequential permutation generation strategy was used. However, this approach was subject to spending long time periods generating undesirable mutants. For some problems, the sequential approach finds solutions very quickly while with others it does not find any solutions within a feasible time period. The random approach was found to more reliably and consistently produce results across a diverse set of mutation problems.

We also initially attempted to develop a mutation generation algorithm that would only generate mutant sequences that satisfy all specified criteria without wasting computation on generating mutants that do not. This approach was abandoned because it was both difficult to make such algorithms and they would require the development of new mutation generation algorithms for every set of criteria. As such, we decided it was preferable to keep separate the concepts of mutant sequence generation and criteria selection, thus enabling the user to specify diverse mutation criteria. For projects requiring disruption of a large number of target sites and having relatively simple criteria, it may be preferable to devise a mutation generation algorithm that does not require the additional filtering step. An example of simple criteria is: mutations must introduce two nucleotide substitutions per site using any of the four nucleotides. Whereas an example of more complex requirements is: mutations must introduce at least two nucleotide substitutions, nucleotide substitutions must be adjacent and must not contain guanosine. However, if disruption of one or a few sites is desired, these inefficiencies do not appreciably impact processing speed.

### Testing the ImiRP target site predictor and mutation generator

To determine whether ImiRP could accurately predict miRNA target sites, we input 150 nucleotide segments from human, mouse and fly 3’UTR sequences and compared output predictions for wild type sequences to predictions generated by TargetScan [3] and PITA [23] for the same sequences (Additional file 1: Figure S4). All 3’UTR sequence data was collected using the University of California, Santa Cruz (UCSC) genome browser [36]. Care was taken when making comparisons to PITA’s predictions. As of the first release of ImiRP, PITA’s database of mature miRNA sequences, obtained from miRBase version 11.0, is out of date. Additionally, PITA’s seed site prediction logic is not based on the currently accepted seed site classification [12]. Consequently, mature miRNA sequences available through miRBase were used to verify PITA’s seed site type predictions. Our results show that ImiRP is capable of accurately predicting 8mer, 7mer-m8, 7mer-A1 and 6mer seed sites. One limitation of these tests is that TargetScan and PITA do not recognize OS-6mer seed sites. However, manual comparison between ImiRP’s OS-6mer seed predictions and mature miRNA sequences in miRBase reveals that ImiRP is capable of correctly predicting OS-6mer seed sites (data not shown). Target site prediction output for three sample 3’UTR sequences is provided in Additional file 1: Figure S4.

To test the program’s capacity to successfully introduce mutations into many sites simultaneously, while also ensuring that new predicted target sites for other miRNAs were not created, we used the aforementioned 150 nucleotide 3’UTR sequences and target site predictions as input into ImiRP. We compared the ImiRP’s target site prediction output between the wild type and mutant sequences to ensure that specified target sites were successfully mutated and that new sites were not created. Sample output for a 150 nucleotide segment from mouse *Pax6* 3’UTR containing six predicted target sites is shown in Additional file 1: Figure S5.

### Software limitations

Though ImiRP is capable of identifying predicted illegitimate miRNA target sites created following mutation of existing target sites, this software does not identify mRNA regulatory elements required for non-miRNA processes. For example, the process of introducing mutations into a 3’UTR sequence could create an RNA-binding protein motif or abolish a pre-existing polyadenylation signal. A freely available web application called Transterm could be used to address this shortcoming [37]. Transterm can identify protein binding sites in mRNA 3’UTRs, and thus a user could compare Transterm results between their original input and mutant 3’UTR sequences to ensure that no known motifs had been created or disrupted. In future versions of ImiRP, we may implement a tool that identifies known polyadenylation signal motifs, and ensures that polyadenylation signals present in the input sequence are not disrupted and new polyadenylation signals are not created following mutation.

One limitation associated with identifying only five classes of target seed site is that other seed site types have been identified that may also be functional. In vivo reporter assays suggest that 4mer and 5mer seed sites also having extensive complementarity to the miRNA 3’ end can be functional [28]. However other studies investigating miRNA-dependent repression in vivo demonstrate that 5mer seed matches, complementary to miRNA positions 2-6, could not mediate repression [38]. As a consequence, we designed the ImiRP target site predictor such that it does not identify 4mer or 5mer target site types. Genome-wide miRNA-mRNA interaction maps from mouse brain generated by Argonaute High-Throughput Sequencing of RNA isolated by crosslinking immunoprecipitation (Ago HITS-CLIP) also suggest that two additional target site types may exist. A third type of 6mer target site, 6merα (complementary to miRNA position 1-6), is capable of binding Ago-miRNA [39]. Additionally, miRNA recognition elements containing G nucleotide bulges at positions 5-6 in the mRNA (G-bulge sites) are evolutionarily conserved and functional in vivo [38]. ImiRP is currently incapable of identifying 6merα and G-bulge target sites and, as a consequence, these site types may be created upon mutation of existing sites. Based on user demand, we may choose to enable identification of 6merα and G-bulge sites though the ImiRP target site predictor.

Often, experiments designed to disrupt a specific sequence motif remove the sequence by deletion as opposed to mutating several nucleotides. The software could be designed to permit deletion mutations as well, however this would significantly complicate illegitimate target site identification. ImiRP compares target site predictions between the input and mutant sequences. Any predicted target sites that are shared between the input and mutant sequences, i.e. target sites for the same miRNA, located at the same position within the sequence, are not considered illegitimate. Information about the position of a predicted target site within the sequence is critical for identifying legitimate versus illegitimate target sites. Enabling deletion mutations will require a more complicated illegitimate target site identification algorithm. We plan to enable the use of insertion and deletion mutations as part of ImiRP’s mutagenesis strategies in future versions. For the time being, users can download the ImiRP source code and modify it to enable insertion/deletion mutations.

Unfortunately, it is challenging to predict with certainty whether a miRNA target site has successfully been abolished by mutation without experimentation. Based on previous experimental evidence [28], introducing at least two nucleotide changes into the mRNA region complementary to miRNA positions 2-7 is likely to abolish regulation by the targeting miRNA. Similarly, predicting the functionality of a newly created miRNA target sites is also challenging without experimental validation. For example, a known *lys-6* site from the *cog-1* 3’UTR in *C. elegans* is often nonfunctional when transplanted into different 3’UTR contexts [35]. ImiRP provides information about all illegitimate target sites created in the output mutant sequence. Available databases documenting validated miRNA-target interactions [40–42] and known miRNA expression profiles [43–45] could be used to assist in determining whether illegitimate target site predictions are likely to occur.

## Conclusion

In summary, we have described ImiRP, software that enables mutation of one or more predicted miRNA target sites within a sequence of interest while ensuring that new predicted target sites for other miRNAs are not created in the process. The ImiRP input interface allows the user to input a sequence of interest and specify the location of one or more target sites to mutate, species of interest, invalid target site types, and mutation strategy. The output interface allows the user to select a desired mutant for each independent region, and view a finalized mutant sequence. Upon completion, the assembled mutant sequence, and miRNA target site predictions for the input and mutant sequences are made available for download. ImiRP source code is accessible open source through GitHub (https://github.com/imirp) under the Apache Version 2 license, and is available as a web application at imirp.org.

### Ethics approval and consent to participate

Not applicable.

### Consent for publication

Not applicable.

## Availability of data and materials

**Project name:** ImiRP

**Project homepage:** imirp.org

**Operating system(s):** Windows, OSX, Linux

**Programming language:** Java, Javascript

**Other requirements:** MongoDB, Java7, SBT

**License:** MIT

## Additional file

Additional file 1: Supplementary Material. (PDF 2901 kb)

## Abbreviations

3’UTR: 3’ untranslated region
6mer: miRNA target site complementary to miRNA positions 2-7
7mer-A1: miRNA target site complementary to miRNA positions 2-7 having an A across from miRNA position 1
7mer-m8: miRNA target site complementary to miRNA position 2-8
8mer: miRNA target site complementary to miRNA positions 2-8 and having an A across from miRNA position 1
ImiRP: Illegitimate miRNA Predictor
miRNA: microRNA
mRNA: messenger RNA
OS-6mer: offset-6mer, miRNA target site complementary to miRNA positions 3-8
PITA: Probability of Interaction by Target Site Accessibility
