# Supplementary material for: ImiRP: a computational approach to microRNA target site mutation
Source: BMC Bioinformatics. 2016 Apr 27;17:190. doi: 10.1186/s12859-016-1057-y (PMC4848830; doi:10.1186/s12859-016-1057-y)
Supplement: Supplementary file 1 — Supplementary Material. (PDF 2901 kb) [file 12859_2016_1057_MOESM1_ESM.pdf]

Supplementary Figures

Supplementary Figure 1. The number of mutant sequences that can be produced using various mutation-generation strategies.

1a)

$$Total\ Mutant\ Sequences = \left[\sum_{i=2}^{N} \binom{N}{i} (n-1)^i\right]^x$$

$$= \left[\sum_{i=2}^{6} \binom{6}{i} (4-1)^i\right]^3$$

$$= \left[\binom{6}{2}3^2 + \binom{6}{3}3^3 + \binom{6}{4}3^4 + \binom{6}{5}3^5 + \binom{6}{6}3^6\right]^3$$

$$\approx 6.8 \times 10^{10}$$

1b)

$$Total\ Mutant\ Sequences = \left[\binom{N-1}{1} (n-1)^2\right]^x$$

$$= \left[(6-1)(4-1)^2\right]^3$$

$$\approx 9.1 \times 10^4$$

1c)

$$Total\ Mutant\ Sequences = \left[\left[\binom{N-1}{1}(n-1)^2\right]^x\right]^y$$

$$= \left[\left[(6-1)(4-1)^2\right]^3\right]^2$$

$$\approx 8.3 \times 10^9$$

1d)

$$Total\ Mutant\ Sequences = \left[\left[\binom{N-1}{1}(n-1)^2\right]^x\right] \times y$$

$$= \left[\left[(6-1)(4-1)^2\right]^3\right] \times 2$$

$$\approx 1.5 \times 10^5$$

Supplementary Figure 1. The number of mutant sequences that can be produced using various mutation-generation strategies.

"N" represents the number of nucleotide positions to mutate per predicted target site. ImiRP introduces mutations only into the 6mer core seed region of each predicted target site. The number of the four nucleotide bases to use for mutagenesis is represented by "n". "x" and "y" designate the number of specified target sites per independent region and the number of independent regions, respectively. **a)** The number of possible mutant sequences produced when at least two nucleotide changes are introduced per target site using any permutation of the four nucleotide bases. In this example, three non-overlapping target sites are present in a single independent region, and the total possible mutants are greater than 60 billion. **b)** When exactly two adjacent nucleotide changes are introduced per site, using any of the four nucleotide bases to generate mutations, the number of possible mutants for a single independent region containing three non-overlapping sites is reduced to approximately 90,000. **c)** If two independent regions containing three target sites each are mutated simultaneously by introducing two adjacent nucleotide changes using any of the four nucleotide bases, over 8 billion mutant sequences are possible. **d)** However, mutating predicted target sites spaced at a distance of seven or more nucleotides apart independent of one another significantly reduces the number of redundant mutations generated. When two independent regions containing three non-overlapping target sites each are mutated in isolation, the number of mutant sequences that need to be processed is reduced to 180,000

```
do:
    copy input_sequence to mutant
    for each site in mutationites:
        for each position in site:
            set nuc to select_random(allowable_nucleotides)
            overwrite mutant[position] to nuc
        end
    end
    if mutant acceptable:
        output mutant to callback
    end
while(further mutations are needed)
```

Supplementary Figure 2.

Pseudocode for ImiRP’s mutation generation algorithm.

```
for each five_prime in mirbase:
        do:
                set sequence_chunk to sequence[window_start, window_end]
                set match_result to check_conditions(five_prime, sequence_chunk)
                increment window_start
                increment window_end
        while(window_end < sequence_end)
end

function check_conditions(five_prime, sequence_chunk):

end
```

Supplementary Figure 3.

Pseudocode for ImiRP's microRNA target site prediction algorithm.

1a) 150 nucleotide segment of human *PAX6* 3'UTR

GTTTTGAAGTTGTTCACAACTACATATCAAAATTAACCATTGTTGATTGT
AAAAAACCATGCCAAAGCCTTTGTATTTCCTTTATTATACAGTTTTCTTT
TTAACCTTATAGTGTGGTGTTACAAATTTTATTTCCATGTTAGATCAACA

Start Position	miRNA	Site Type	Predicted by
22	hsa-miR-190a/b	8mer	TS
35	hsa-miR-140-5p	7mer-m8+G:U	PITA
60	hsa-miR-96/182/507/ 1271	7mer-A1	TS

Summary of ImiRP predictions for wild type human *PAX6* sequence:

position	mirna	type	gu
2	>hsa-miR-616-5p MIMAT0003284 Homo sapiens	OS-6mer	no
6	>hsa-miR-513c-3p MIMAT0022728 Homo sapiens	OS-6mer	yes
14	>hsa-miR-758-3p MIMAT0003879 Homo sapiens	6mer	no
16	>hsa-miR-382-5p MIMAT0000737 Homo sapiens	OS-6mer	no
16	>hsa-miR-494-5p MIMAT0026607 Homo sapiens	7mer-A1	yes
17	>hsa-miR-758-5p MIMAT0022929 Homo sapiens	OS-6mer	yes
18	>hsa-miR-203a-5p MIMAT0031890 Homo sapiens	7mer-A1	yes
18	>hsa-miR-140-5p MIMAT0000431 Homo sapiens	OS-6mer	yes
18	>hsa-miR-876-3p MIMAT0004925 Homo sapiens	OS-6mer	yes
19	>hsa-miR-20b-3p MIMAT0004752 Homo sapiens	OS-6mer	no
20	>hsa-miR-299-3p MIMAT0000687 Homo sapiens	7mer-A1	yes
**22**	**>hsa-miR-190a-5p MIMAT0000458 Homo sapiens**	**8mer**	**no**
23	>hsa-miR-576-3p MIMAT0004796 Homo sapiens	OS-6mer	yes
26	>hsa-miR-9-5p MIMAT0000441 Homo sapiens	OS-6mer	yes
28	>hsa-miR-19a-5p MIMAT0004490 Homo sapiens	6mer	yes
28	>hsa-miR-19b-1-5p MIMAT0004491 Homo sapiens	6mer	yes
28	>hsa-miR-19b-2-5p MIMAT0004492 Homo sapiens	6mer	yes
29	>hsa-miR-590-3p MIMAT0004801 Homo sapiens	7mer-A1	no
30	>hsa-miR-29a-5p MIMAT0004503 Homo sapiens	OS-6mer	yes
34	>hsa-miR-487b-5p MIMAT0026614 Homo sapiens	6mer	no
34	>hsa-miR-758-5p MIMAT0022929 Homo sapiens	7mer-m8	yes
35	>hsa-miR-203a-5p MIMAT0031890 Homo sapiens	6mer	yes
**35**	**>hsa-miR-140-5p MIMAT0000431 Homo sapiens**	**7mer-m8**	**yes**
35	>hsa-miR-584-5p MIMAT0003249 Homo sapiens	OS-6mer	no
35	>hsa-miR-876-3p MIMAT0004925 Homo sapiens	OS-6mer	yes
37	>hsa-miR-194-3p MIMAT0004671 Homo sapiens	6mer	yes
38	>hsa-miR-106a-3p MIMAT0004517 Homo sapiens	OS-6mer	yes

38	>hsa-miR-128-3p MIMAT0000424 Homo sapiens	OS-6mer	yes
39	>hsa-miR-132-3p MIMAT0000426 Homo sapiens	6mer	yes
40	>hsa-miR-545-3p MIMAT0003165 Homo sapiens	7mer-A1	yes
40	>hsa-miR-3065-5p MIMAT0015066 Homo sapiens	7mer-A1	no
41	>hsa-miR-302c-5p MIMAT0000716 Homo sapiens	6mer	yes
41	>hsa-miR-505-3p MIMAT0002876 Homo sapiens	7mer-m8	yes
42	>hsa-miR-34a-3p MIMAT0004557 Homo sapiens	7mer-m8	yes
42	>hsa-miR-369-5p MIMAT0001621 Homo sapiens	OS-6mer	yes
43	>hsa-miR-551b-5p MIMAT0004794 Homo sapiens	OS-6mer	no
45	>hsa-miR-132-3p MIMAT0000426 Homo sapiens	OS-6mer	yes
46	>hsa-miR-106a-3p MIMAT0004517 Homo sapiens	7mer-A1	yes
46	>hsa-miR-139-5p MIMAT0000250 Homo sapiens	OS-6mer	yes
46	>hsa-miR-374a-5p MIMAT0000727 Homo sapiens	7mer-A1	yes
46	>hsa-miR-374b-5p MIMAT0004955 Homo sapiens	7mer-A1	yes
47	>hsa-miR-450b-5p MIMAT0004909 Homo sapiens	8mer	yes
49	>hsa-miR-19a-5p MIMAT0004490 Homo sapiens	OS-6mer	yes
49	>hsa-miR-19b-1-5p MIMAT0004491 Homo sapiens	OS-6mer	yes
49	>hsa-miR-19b-2-5p MIMAT0004492 Homo sapiens	OS-6mer	yes
49	>hsa-miR-129-5p MIMAT0000242 Homo sapiens	8mer	yes
53	>hsa-miR-329-5p MIMAT0026555 Homo sapiens	OS-6mer	no
54	>hsa-miR-29b-1-5p MIMAT0004514 Homo sapiens	OS-6mer	no
55	>hsa-miR-203a-5p MIMAT0031890 Homo sapiens	6mer	yes
55	>hsa-miR-140-5p MIMAT0000431 Homo sapiens	OS-6mer	yes
55	>hsa-miR-584-5p MIMAT0003249 Homo sapiens	7mer-m8	yes
55	>hsa-miR-758-5p MIMAT0022929 Homo sapiens	6mer	no
55	>hsa-miR-876-3p MIMAT0004925 Homo sapiens	OS-6mer	yes
**60**	**>hsa-miR-96-5p MIMAT0000095 Homo sapiens**	**7mer-A1**	**no**
**60**	**>hsa-miR-182-5p MIMAT0000259 Homo sapiens**	**7mer-A1**	**no**
**60**	**>hsa-miR-1271-5p MIMAT0005796 Homo sapiens**	**7mer-A1**	**no**
61	>hsa-miR-9-5p MIMAT0000441 Homo sapiens	7mer-m8	yes
63	>hsa-miR-19a-5p MIMAT0004490 Homo sapiens	6mer	yes
63	>hsa-miR-19b-1-5p MIMAT0004491 Homo sapiens	6mer	yes
63	>hsa-miR-19b-2-5p MIMAT0004492 Homo sapiens	6mer	yes
64	>hsa-miR-329-5p MIMAT0026555 Homo sapiens	7mer-m8	yes
65	>hsa-miR-27a-5p MIMAT0004501 Homo sapiens	6mer	yes
65	>hsa-miR-3158-3p MIMAT0015032 Homo sapiens	7mer-m8	yes
66	>hsa-miR-485-5p MIMAT0002175 Homo sapiens	6mer	yes
69	>hsa-miR-340-5p MIMAT0004692 Homo sapiens	7mer-m8	yes
69	>hsa-miR-624-3p MIMAT0004807 Homo sapiens	7mer-A1	yes
70	>hsa-miR-19a-3p MIMAT0000073 Homo sapiens	OS-6mer	yes
70	>hsa-miR-19b-3p MIMAT0000074 Homo sapiens	OS-6mer	yes

a) 150 nucleotide segment of mouse *Pax6* 3'UTR

AAAAACTATCTGTTGGTTTTCCAAAGGTTGTTAACAGATGAAGTTTATGT
GCAAAAAAGGGTAAGATATGAATTCAAGGAGAAGTTGATAGCTAAAAGGT
AGAGTGTGTCTTCGATATAATACAATTTGTTTTATGTCAAAATGTAAGTA

Start Position	miRNA	Site Type	Predicted by
16	mmu-miR-7a/b	8mer+G:U	PITA
21	mmu-miR-9	6mer	PITA
44	mmu-miR-376c	7mer-m8+G:U	PITA
50	mmu-miR-450b-5p	7mer-A1	PITA
51	mmu-miR-129-5p	8mer	TS, PITA
104	mmu-miR-329	6mer	PITA

Summary of ImiRP predictions for wild type mouse *Pax6* sequence:

position	mirna	type	gu
2	>mmu-miR-19a-5p MIMAT0004660 Mus musculus	7mer-A1	no
3	>mmu-miR-29b-1-5p MIMAT0004523 Mus musculus	OS-6mer	yes
3	>mmu-miR-29b-2-5p MIMAT0017063 Mus musculus	6mer	yes
4	>mmu-miR-218-2-3p MIMAT0005444 Mus musculus	6mer	yes
5	>mmu-miR-196a-5p MIMAT0000518 Mus musculus	7mer-m8	yes
5	>mmu-miR-196b-5p MIMAT0001081 Mus musculus	7mer-m8	yes
6	>mmu-let-7g-5p MIMAT0000121 Mus musculus	OS-6mer	yes
6	>mmu-let-7i-5p MIMAT0000122 Mus musculus	OS-6mer	yes
6	>mmu-let-7d-5p MIMAT0000383 Mus musculus	OS-6mer	yes
6	>mmu-let-7a-5p MIMAT0000521 Mus musculus	OS-6mer	yes
6	>mmu-let-7b-5p MIMAT0000522 Mus musculus	OS-6mer	yes
6	>mmu-let-7c-5p MIMAT0000523 Mus musculus	OS-6mer	yes
6	>mmu-let-7e-5p MIMAT0000524 Mus musculus	OS-6mer	yes
6	>mmu-let-7f-5p MIMAT0000525 Mus musculus	OS-6mer	yes
6	>mmu-miR-98-5p MIMAT0000545 Mus musculus	OS-6mer	yes
6	>mmu-miR-1839-5p MIMAT0009456 Mus musculus	6mer	yes
7	>mmu-miR-488-5p MIMAT0003449 Mus musculus	OS-6mer	no
9	>mmu-miR-191-5p MIMAT0000221 Mus musculus	6mer	yes
10	>mmu-miR-194-5p MIMAT0000224 Mus musculus	OS-6mer	yes
10	>mmu-miR-196b-3p MIMAT0017170 Mus musculus	6mer	yes
10	>mmu-miR-421-3p MIMAT0004869 Mus musculus	7mer-m8	yes
11	>mmu-miR-16-2-3p MIMAT0017018 Mus musculus	6mer	yes
11	>mmu-miR-338-3p MIMAT0000582 Mus musculus	6mer	yes
11	>mmu-miR-505-3p MIMAT0003513 Mus musculus	OS-6mer	yes

11	>mmu-miR-3098-5p MIMAT0014917 Mus musculus	OS-6mer	yes
14	>mmu-miR-497a-3p MIMAT0017247 Mus musculus	6mer	no
14	>mmu-miR-742-3p MIMAT0004237 Mus musculus	7mer-m8	yes
**16**	**>mmu-miR-7a-5p MIMAT0000677 Mus musculus**	**8mer**	**yes**
17	>mmu-miR-1958 MIMAT0009431 Mus musculus	OS-6mer	yes
18	>mmu-miR-204-3p MIMAT0017002 Mus musculus	OS-6mer	yes
18	>mmu-miR-450a-1-3p MIMAT0017182 Mus musculus	8mer	yes
18	>mmu-miR-450b-3p MIMAT0003512 Mus musculus	8mer	yes
19	>mmu-miR-3057-5p MIMAT0014822 Mus musculus	7mer-A1	yes
**21**	**>mmu-miR-9-5p MIMAT0000142 Mus musculus**	**6mer**	**no**
23	>mmu-miR-511-5p MIMAT0004940 Mus musculus	OS-6mer	yes
23	>mmu-miR-3061-3p MIMAT0014829 Mus musculus	OS-6mer	no
24	>mmu-miR-1264-3p MIMAT0014803 Mus musculus	OS-6mer	yes
27	>mmu-miR-196a-1-3p MIMAT0017013 Mus musculus	7mer-A1	yes
27	>mmu-miR-21a-3p MIMAT0004628 Mus musculus	7mer-A1	yes
28	>mmu-miR-194-5p MIMAT0000224 Mus musculus	OS-6mer	yes
30	>mmu-miR-758-5p MIMAT0017235 Mus musculus	OS-6mer	yes
32	>mmu-miR-544-5p MIMAT0017282 Mus musculus	OS-6mer	yes
38	>mmu-miR-205-5p MIMAT0000238 Mus musculus	OS-6mer	no
38	>mmu-miR-335-3p MIMAT0004704 Mus musculus	OS-6mer	yes
39	>mmu-miR-205-3p MIMAT0017003 Mus musculus	6mer	yes
40	>mmu-miR-3099-5p MIMAT0014815 Mus musculus	OS-6mer	yes
40	>mmu-miR-1964-3p MIMAT0009437 Mus musculus	OS-6mer	yes
41	>mmu-miR-100-3p MIMAT0017051 Mus musculus	OS-6mer	yes
42	>mmu-miR-151-3p MIMAT0000161 Mus musculus	6mer	yes
42	>mmu-miR-345-3p MIMAT0004656 Mus musculus	OS-6mer	yes
43	>mmu-miR-30a-5p MIMAT0000128 Mus musculus	6mer	yes
43	>mmu-miR-30b-5p MIMAT0000130 Mus musculus	6mer	yes
43	>mmu-miR-30e-5p MIMAT0000248 Mus musculus	6mer	yes
43	>mmu-miR-30c-5p MIMAT0000514 Mus musculus	6mer	yes
43	>mmu-miR-30d-5p MIMAT0000515 Mus musculus	6mer	yes
43	>mmu-miR-384-5p MIMAT0004745 Mus musculus	6mer	yes
43	>mmu-miR-463-3p MIMAT0004758 Mus musculus	OS-6mer	yes
44	>mmu-miR-340-5p MIMAT0004651 Mus musculus	6mer	yes
44	>mmu-miR-376b-3p MIMAT0001092 Mus musculus	OS-6mer	yes
**44**	**>mmu-miR-376c-3p MIMAT0003183 Mus musculus**	**7mer-m8**	**yes**
45	>mmu-miR-322-3p MIMAT0000549 Mus musculus	OS-6mer	yes
45	>mmu-miR-410-3p MIMAT0001091 Mus musculus	6mer	yes
45	>mmu-miR-344d-3p MIMAT0014808 Mus musculus	6mer	yes
46	>mmu-miR-466b-3p MIMAT0004876 Mus musculus	6mer	yes
46	>mmu-miR-466c-3p MIMAT0004878 Mus musculus	6mer	yes

46	>mmu-miR-466e-3p MIMAT0004880 Mus musculus	OS-6mer	yes
46	>mmu-miR-669d-3p MIMAT0017324 Mus musculus	OS-6mer	yes
47	>mmu-miR-448-3p MIMAT0001533 Mus musculus	8mer	yes
48	>mmu-let-7g-3p MIMAT0004519 Mus musculus	OS-6mer	yes
48	>mmu-let-7i-3p MIMAT0004520 Mus musculus	7mer-A1	yes
48	>mmu-let-7a-2-3p MIMAT0017015 Mus musculus	7mer-A1	yes
48	>mmu-let-7c-1-3p MIMAT0004622 Mus musculus	7mer-A1	yes
49	>mmu-miR-92a-3p MIMAT0000539 Mus musculus	OS-6mer	no
49	>mmu-miR-25-3p MIMAT0000652 Mus musculus	OS-6mer	no
49	>mmu-miR-32-5p MIMAT0000654 Mus musculus	OS-6mer	no
49	>mmu-miR-363-3p MIMAT0000708 Mus musculus	OS-6mer	no
49	>mmu-miR-92b-3p MIMAT0004899 Mus musculus	OS-6mer	no
50	>mmu-miR-450a-5p MIMAT0001546 Mus musculus	7mer-A1	yes
50	>mmu-miR-452-5p MIMAT0001637 Mus musculus	OS-6mer	no
**50**	**>mmu-miR-450b-5p MIMAT0003511 Mus musculus**	**7mer-A1**	**no**
**51**	**>mmu-miR-129-5p MIMAT0000209 Mus musculus**	**8mer**	**no**
51	>mmu-miR-19b-1-5p MIMAT0017065 Mus musculus	OS-6mer	no
51	>mmu-miR-669b-5p MIMAT0003476 Mus musculus	OS-6mer	yes
55	>mmu-miR-130a-5p MIMAT0016983 Mus musculus	7mer-m8	yes
56	>mmu-miR-204-5p MIMAT0000237 Mus musculus	OS-6mer	no
56	>mmu-miR-130b-5p MIMAT0004583 Mus musculus	6mer	yes
56	>mmu-miR-211-5p MIMAT0000668 Mus musculus	OS-6mer	no
57	>mmu-miR-129-1-3p MIMAT0016994 Mus musculus	OS-6mer	yes
57	>mmu-miR-129-2-3p MIMAT0000544 Mus musculus	OS-6mer	yes
57	>mmu-miR-362-5p MIMAT0000706 Mus musculus	7mer-A1	yes
57	>mmu-miR-1249-3p MIMAT0010560 Mus musculus	OS-6mer	yes
57	>mmu-miR-500-5p MIMAT0017258 Mus musculus	7mer-A1	yes
57	>mmu-miR-3061-3p MIMAT0014829 Mus musculus	8mer	yes
58	>mmu-miR-18a-3p MIMAT0004626 Mus musculus	OS-6mer	yes
58	>mmu-miR-874-3p MIMAT0004853 Mus musculus	7mer-A1	yes
60	>mmu-miR-429-5p MIMAT0017178 Mus musculus	7mer-m8	no
61	>mmu-miR-200b-5p MIMAT0004545 Mus musculus	8mer	no
61	>mmu-miR-200a-5p MIMAT0004619 Mus musculus	8mer	no
61	>mmu-miR-200c-5p MIMAT0004663 Mus musculus	8mer	yes
61	>mmu-miR-431-5p MIMAT0001418 Mus musculus	OS-6mer	yes
62	>mmu-miR-1941-3p MIMAT0009406 Mus musculus	7mer-A1	no
69	>mmu-miR-10a-3p MIMAT0004659 Mus musculus	OS-6mer	yes
69	>mmu-miR-32-3p MIMAT0017050 Mus musculus	6mer	yes
71	>mmu-miR-183-3p MIMAT0004539 Mus musculus	7mer-A1	no
71	>mmu-miR-3068-3p MIMAT0014843 Mus musculus	OS-6mer	no
73	>mmu-miR-470-5p MIMAT0002111 Mus musculus	OS-6mer	yes

73	>mmu-miR-547-5p MIMAT0017210 Mus musculus	OS-6mer	yes
74	>mmu-miR-212-5p MIMAT0017053 Mus musculus	7mer-A1	yes
74	>mmu-miR-3075-3p MIMAT0014859 Mus musculus	7mer-m8	yes
75	>mmu-miR-362-5p MIMAT0000706 Mus musculus	OS-6mer	no
75	>mmu-miR-500-5p MIMAT0017258 Mus musculus	OS-6mer	no
76	>mmu-miR-130a-5p MIMAT0016983 Mus musculus	7mer-A1	yes
76	>mmu-miR-130b-5p MIMAT0004583 Mus musculus	7mer-A1	yes
76	>mmu-miR-103-1-5p MIMAT0017024 Mus musculus	OS-6mer	yes
76	>mmu-miR-103-2-5p MIMAT0017025 Mus musculus	OS-6mer	yes
76	>mmu-miR-107-5p MIMAT0017048 Mus musculus	OS-6mer	yes
78	>mmu-miR-22-5p MIMAT0004629 Mus musculus	OS-6mer	yes
79	>mmu-miR-103-1-5p MIMAT0017024 Mus musculus	OS-6mer	yes
79	>mmu-miR-103-2-5p MIMAT0017025 Mus musculus	OS-6mer	yes
79	>mmu-miR-107-5p MIMAT0017048 Mus musculus	OS-6mer	yes
79	>mmu-miR-320-5p MIMAT0017057 Mus musculus	OS-6mer	no
81	>mmu-miR-3099-5p MIMAT0014815 Mus musculus	8mer	yes
81	>mmu-miR-1964-3p MIMAT0009437 Mus musculus	8mer	yes
83	>mmu-miR-434-5p MIMAT0001421 Mus musculus	OS-6mer	yes
84	>mmu-miR-34a-3p MIMAT0017022 Mus musculus	7mer-A1	yes
84	>mmu-miR-369-5p MIMAT0003185 Mus musculus	OS-6mer	yes
84	>mmu-miR-505-3p MIMAT0003513 Mus musculus	7mer-A1	yes
85	>mmu-miR-361-5p MIMAT0000704 Mus musculus	6mer	yes
85	>mmu-miR-3074-1-3p MIMAT0014857 Mus musculus	OS-6mer	yes
86	>mmu-miR-215-3p MIMAT0017169 Mus musculus	6mer	yes
88	>mmu-miR-101a-5p MIMAT0004526 Mus musculus	OS-6mer	yes
94	>mmu-miR-130a-5p MIMAT0016983 Mus musculus	OS-6mer	yes
95	>mmu-miR-511-5p MIMAT0004940 Mus musculus	7mer-m8	yes
95	>mmu-miR-3061-3p MIMAT0014829 Mus musculus	7mer-m8	no
96	>mmu-miR-532-5p MIMAT0002889 Mus musculus	OS-6mer	yes
97	>mmu-miR-3106-3p MIMAT0014818 Mus musculus	7mer-A1	yes
97	>mmu-miR-3110-5p MIMAT0014951 Mus musculus	7mer-m8	yes
98	>mmu-miR-346-5p MIMAT0000597 Mus musculus	OS-6mer	yes
99	>mmu-miR-676-5p MIMAT0003781 Mus musculus	6mer	no
102	>mmu-miR-181a-5p MIMAT0000210 Mus musculus	6mer	yes
102	>mmu-miR-181c-5p MIMAT0000674 Mus musculus	6mer	yes
102	>mmu-miR-181b-5p MIMAT0000673 Mus musculus	6mer	yes
102	>mmu-miR-543-3p MIMAT0003168 Mus musculus	OS-6mer	yes
102	>mmu-miR-181d-5p MIMAT0004324 Mus musculus	6mer	yes
102	>mmu-miR-574-3p MIMAT0004894 Mus musculus	6mer	yes
102	>mmu-miR-3572-3p MIMAT0020636 Mus musculus	6mer	yes
103	>mmu-miR-23b-3p MIMAT0000125 Mus musculus	OS-6mer	yes

103	>mmu-miR-106b-3p MIMAT0004582 Mus musculus	6mer	yes
103	>mmu-miR-23a-3p MIMAT0000532 Mus musculus	OS-6mer	yes
103	>mmu-miR-1b-5p MIMAT0005835 Mus musculus	7mer-m8	yes
**104**	**>mmu-miR-329-3p MIMAT0000567 Mus musculus**	**6mer**	**no**
104	>mmu-miR-362-3p MIMAT0004684 Mus musculus	6mer	no
104	>mmu-miR-466b-3p MIMAT0004876 Mus musculus	OS-6mer	yes
104	>mmu-miR-466c-3p MIMAT0004878 Mus musculus	OS-6mer	yes
104	>mmu-miR-466m-3p MIMAT0014883 Mus musculus	6mer	yes
104	>mmu-miR-466o-3p MIMAT0014886 Mus musculus	6mer	yes
106	>mmu-miR-124-3p MIMAT0000134 Mus musculus	7mer-m8	yes
106	>mmu-miR-3095-3p MIMAT0014912 Mus musculus	6mer	yes
107	>mmu-miR-208a-3p MIMAT0000520 Mus musculus	OS-6mer	yes
107	>mmu-miR-743a-3p MIMAT0004238 Mus musculus	OS-6mer	no
107	>mmu-miR-743b-3p MIMAT0004840 Mus musculus	OS-6mer	no
107	>mmu-miR-208b-3p MIMAT0004939 Mus musculus	OS-6mer	yes
107	>mmu-miR-6540-5p MIMAT0025585 Mus musculus	OS-6mer	yes
108	>mmu-miR-7a-5p MIMAT0000677 Mus musculus	OS-6mer	no
109	>mmu-miR-151-5p MIMAT0004536 Mus musculus	7mer-A1	yes
109	>mmu-miR-802-3p MIMAT0017240 Mus musculus	7mer-A1	yes
112	>mmu-miR-325-3p MIMAT0004640 Mus musculus	OS-6mer	yes
118	>mmu-miR-503-3p MIMAT0004790 Mus musculus	OS-6mer	yes
119	>mmu-miR-223-5p MIMAT0017056 Mus musculus	OS-6mer	no
119	>mmu-miR-1198-5p MIMAT0005859 Mus musculus	OS-6mer	yes

1c) 150 nucleotide segment of fly *Ey* 3'UTR

```
TATCTAAAGGAGGCCATAATAAATCGAATTTACATATCTCTTGAAAAATA
ATGGAGGTTGTAGAAAAATACATTTGTATGTATAAATTATATAGTTCCGC
CCATTAAATCCAATCTATAGTGTAGAATAATTGGTGTAAATTAAATGATA
```

Start Position	miRNA	Site Type	Predicted by
16	dme-miR-1000	7mer-A1+G:U	PITA
26	dme-miR-1014	7mer-A1	TS
32	dme-miR-190	7mer-m8	TS
34	dme-miR-977	7mer-m8	TS

Summary of ImiRP predictions for wild type fly *Ey* sequence:

position	mirna	type	gu
1	>dme-miR-983-3p MIMAT0020880 Drosophila melanogaster	8mer	yes
2	>dme-miR-303-5p MIMAT0000388 Drosophila melanogaster	7mer-A1	yes
4	>dme-miR-9a-5p MIMAT0000114 Drosophila melanogaster	6mer	yes
4	>dme-miR-79-5p MIMAT0020814 Drosophila melanogaster	6mer	yes
4	>dme-miR-9c-5p MIMAT0000392 Drosophila melanogaster	6mer	yes
4	>dme-miR-9b-5p MIMAT0000395 Drosophila melanogaster	6mer	yes
6	>dme-miR-316-5p MIMAT0000408 Drosophila melanogaster	OS-6mer	yes
9	>dme-miR-282-5p MIMAT0000346 Drosophila melanogaster	OS-6mer	yes
**16**	**>dme-miR-1000-5p MIMAT0005520 Drosophila melanogaster**	**7mer-A1**	**yes**
21	>dme-bantam-5p MIMAT0020823 Drosophila melanogaster	7mer-A1	yes
22	>dme-miR-315-5p MIMAT0000407 Drosophila melanogaster	7mer-m8	yes
24	>dme-miR-314-3p MIMAT0000406 Drosophila melanogaster	6mer	no
25	>dme-miR-10-3p MIMAT0012531 Drosophila melanogaster	8mer	no
25	>dme-miR-1006-3p MIMAT0005019 Drosophila melanogaster	8mer	no
26	>dme-miR-958-3p MIMAT0005471 Drosophila melanogaster	OS-6mer	yes
**26**	**>dme-miR-1014-3p MIMAT0005027 Drosophila melanogaster**	**7mer-A1**	**no**
27	>dme-miR-998-5p MIMAT0020898 Drosophila melanogaster	OS-6mer	yes
28	>dme-miR-4969-5p MIMAT0020195 Drosophila melanogaster	OS-6mer	no
29	>dme-miR-971-5p MIMAT0020868 Drosophila melanogaster	OS-6mer	no
31	>dme-miR-9388-5p MIMAT0035238 Drosophila melanogaster	6mer	no
**32**	**>dme-miR-190-5p MIMAT0005467 Drosophila melanogaster**	**7mer-m8**	**no**
34	>dme-miR-975-3p MIMAT0020872 Drosophila melanogaster	OS-6mer	yes
**34**	**>dme-miR-977-3p MIMAT0005493 Drosophila melanogaster**	**7mer-m8**	**no**
35	>dme-let-7-5p MIMAT0000396 Drosophila melanogaster	6mer	yes
35	>dme-miR-317-5p MIMAT0020843 Drosophila melanogaster	6mer	yes
35	>dme-miR-967-5p MIMAT0005482 Drosophila melanogaster	6mer	no
35	>dme-miR-984-5p MIMAT0005500 Drosophila melanogaster	6mer	yes

37	>dme-miR-281-1-5p MIMAT0000344 Drosophila melanogaster	6mer	no
40	>dme-miR-314-3p MIMAT0000406 Drosophila melanogaster	OS-6mer	yes
40	>dme-miR-315-3p MIMAT0020841 Drosophila melanogaster	8mer	yes
43	>dme-miR-980-5p MIMAT0020877 Drosophila melanogaster	8mer	yes

Supplementary Figure 4. Testing the ImiRP Target Site Predictor

Several 3'UTR segments from three different species were provided as input to ImiRP. ImiRP target site predictions were compared to predictions made by PITA and TargetScan for each sequence. MiRBase was used to validate the ImiRP target site predictions. Three sample test results are provided. a) 150 nucleotide segment of the human *PAX6* 3'UTR: ImiRP successfully identified target sites for miR-190a, miR-140-5p, miR-96, miR-182, and miR-1271. Target sites for miR-190b and miR-507 were not detected because these miRNAs are not high confidence miRNAs. miR-190b has less than five reads mapping to its 3' arm, and miR-507 has no reads mapping to the 5' arm. b) 150 nucleotide segment of the mouse *Pax6* 3'UTR: ImiRP successfully identified predicted target sites for miR-7a, miR-9, miR-376c, miR-450b-5p, miR-129-5p, and miR-329. miR-7b was not detected because this miRNA is not present in the miRBase version 21 high confidence dataset. c) 150 nucleotide segment of the fly *Ey* 3'UTR: ImiRP successfully identified predicted target sites for miR-1000, miR-1014, miR-190, and miR-977.

Supplementary Figure 5. Testing the final mutant sequence validity

2a)

```
Wild type 1 AAAAACTATCTGTTGGTTTTCCAAAGGTTGTTAACAGATGAAGTTTATGT
            |||||||||||||||||   |||   ||||||||||||||||||   |||
Mutant    1 AAAAACTATCTGTTGGTCGCCCAGTAGTTGTTAACAGATGAAGTGGGTGT

         51 GCAAAAAAGGGTAAGATATGAATTCAAGGAGAAGTTGATAGCTAAAAGGT
            ||   |||||||||||||||||||||||||||||||||||||||||||||
         51 GCGTCAAAGGGTAAGATATGAATTCAAGGAGAAGTTGATAGCTAAAAGGT

        101 AGAGTGTGTCTTCGATATAATACAATTTGTTTTATGTCAAAATGTAAGTA
            ||||||   |||||||||||||||||||||||||||||||||||||||||
        101 AGAGTGGACCTTCGATATAATACAATTTGTTTTATGTCAAAATGTAAGTA
```

2b) Comparison of ImiRP target site predictions for the wild type and mutant mouse *Pax6* 3'UTR segment:

	Wild type results			Mutant Results		
position	mirna	type	gu	mirna	type	gu
…						
11	>mmu-miR-16-2-3p	6mer	yes	>mmu-miR-16-2-3p	6mer	yes
11	>mmu-miR-338-3p	6mer	yes	>mmu-miR-338-3p	6mer	yes
11	>mmu-miR-505-3p	OS-6mer	yes	>mmu-miR-505-3p	OS-6mer	yes
11	>mmu-miR-3098-5p	OS-6mer	yes	>mmu-miR-3098-5p	OS-6mer	yes
14	>mmu-miR-497a-3p	6mer	no			
14	>mmu-miR-742-3p	7mer-m8	yes			
16	>mmu-miR-7a-5p	8mer	yes			
17	>mmu-miR-1958	OS-6mer	yes			
18	>mmu-miR-204-3p	OS-6mer	yes			
18	>mmu-miR-450a-1-3p	8mer	yes			
18	>mmu-miR-450b-3p	8mer	yes			
19	>mmu-miR-3057-5p	7mer-A1	yes			
21	>mmu-miR-9-5p	6mer	no			
22				>mmu-miR-93-3p	6mer	yes
22				>mmu-miR-370-3p	OS-6mer	yes
22				>mmu-miR-3098-3p	OS-6mer	yes
23				>mmu-miR-138-1-3p	OS-6mer	yes
23	>mmu-miR-511-5p	OS-6mer	yes			
23	>mmu-miR-3061-3p	OS-6mer	no			

24	>mmu-miR-1264-3p	OS-6mer	yes			
24				>mmu-miR-883a-3p	6mer	yes
24				>mmu-miR-883b-3p	6mer	yes
24				>mmu-miR-872-3p	OS-6mer	yes
26				>mmu-miR-674-3p	6mer	yes
27	>mmu-miR-196a-1-3p	7mer-A1	yes	>mmu-miR-196a-1-3p	7mer-A1	yes
27	>mmu-miR-21a-3p	7mer-A1	yes	>mmu-miR-21a-3p	7mer-A1	yes
28	>mmu-miR-194-5p	OS-6mer	yes	>mmu-miR-194-5p	OS-6mer	yes
30	>mmu-miR-758-5p	OS-6mer	yes	>mmu-miR-758-5p	OS-6mer	yes
32	>mmu-miR-544-5p	OS-6mer	yes	>mmu-miR-544-5p	OS-6mer	yes
38	>mmu-miR-205-5p	OS-6mer	no	>mmu-miR-205-5p	OS-6mer	no
38	>mmu-miR-335-3p	OS-6mer	yes	>mmu-miR-335-3p	OS-6mer	yes
39	>mmu-miR-205-3p	6mer	yes	>mmu-miR-205-3p	6mer	yes
40	>mmu-miR-3099-5p	OS-6mer	yes			
40	>mmu-miR-1964-3p	OS-6mer	yes			
41	>mmu-miR-100-3p	OS-6mer	yes			
42	>mmu-miR-151-3p	6mer	yes			
42	>mmu-miR-345-3p	OS-6mer	yes			
43	>mmu-miR-30a-5p	6mer	yes			
43	>mmu-miR-30b-5p	6mer	yes			
43	>mmu-miR-30e-5p	6mer	yes			
43	>mmu-miR-30c-5p	6mer	yes			
43	>mmu-miR-30d-5p	6mer	yes			
43	>mmu-miR-384-5p	6mer	yes			
43	>mmu-miR-463-3p	OS-6mer	yes			
43				>mmu-miR-742-5p	6mer	yes
43				>mmu-miR-1839-3p	OS-6mer	yes
44	>mmu-miR-340-5p	6mer	yes			
44	>mmu-miR-376b-3p	OS-6mer	yes			
44	>mmu-miR-376c-3p	7mer-m8	yes			
45	>mmu-miR-322-3p	OS-6mer	yes			
45	>mmu-miR-410-3p	6mer	yes			
45	>mmu-miR-344d-3p	6mer	yes			
45				>mmu-miR-448-5p	OS-6mer	yes
45				>mmu-miR-667-3p	OS-6mer	yes
45				>mmu-miR-3093-5p	6mer	yes
46				>mmu-miR-329-3p	OS-6mer	no
46				>mmu-miR-362-3p	OS-6mer	no
46	>mmu-miR-466b-3p	6mer	yes			
46	>mmu-miR-466c-3p	6mer	yes			
46	>mmu-miR-466e-3p	OS-6mer	yes			
46	>mmu-miR-669d-3p	OS-6mer	yes			
47	>mmu-miR-448-3p	8mer	yes			

48	>mmu-let-7g-3p	OS-6mer	yes			
48	>mmu-let-7i-3p	7mer-A1	yes			
48	>mmu-let-7a-2-3p	7mer-A1	yes			
48	>mmu-let-7c-1-3p	7mer-A1	yes			
49	>mmu-miR-92a-3p	OS-6mer	no			
49	>mmu-miR-25-3p	OS-6mer	no			
49	>mmu-miR-32-5p	OS-6mer	no			
49	>mmu-miR-363-3p	OS-6mer	no			
49	>mmu-miR-92b-3p	OS-6mer	no			
49				>mmu-miR-500-3p	6mer	yes
50	>mmu-miR-450a-5p	7mer-A1	yes			
50	>mmu-miR-452-5p	OS-6mer	no			
50	>mmu-miR-450b-5p	7mer-A1	no			
51	>mmu-miR-129-5p	8mer	no			
51	>mmu-miR-19b-1-5p	OS-6mer	no			
51	>mmu-miR-669b-5p	OS-6mer	yes			
51				>mmu-miR-652-3p	OS-6mer	yes
55	>mmu-miR-130a-5p	7mer-m8	yes			
56				>mmu-miR-130a-5p	6mer	yes
56	>mmu-miR-204-5p	OS-6mer	no	>mmu-miR-204-5p	OS-6mer	no
56	>mmu-miR-130b-5p	6mer	yes	>mmu-miR-130b-5p	6mer	yes
56	>mmu-miR-211-5p	OS-6mer	no	>mmu-miR-211-5p	OS-6mer	no
…						
98	>mmu-miR-346-5p	OS-6mer	yes	>mmu-miR-346-5p	OS-6mer	yes
99	>mmu-miR-676-5p	6mer	no	>mmu-miR-676-5p	6mer	no
102	>mmu-miR-181a-5p	6mer	yes			
102	>mmu-miR-181c-5p	6mer	yes			
102	>mmu-miR-181b-5p	6mer	yes			
102	>mmu-miR-543-3p	OS-6mer	yes			
102	>mmu-miR-181d-5p	6mer	yes			
102	>mmu-miR-574-3p	6mer	yes			
102	>mmu-miR-3572-3p	6mer	yes			
102				>mmu-miR-668-3p	OS-6mer	yes
103				>mmu-miR-136-5p	OS-6mer	yes
103	>mmu-miR-23b-3p	OS-6mer	yes			
103	>mmu-miR-106b-3p	6mer	yes			
103	>mmu-miR-23a-3p	OS-6mer	yes			
103	>mmu-miR-1b-5p	7mer-m8	yes			
104	>mmu-miR-329-3p	6mer	no			
104	>mmu-miR-362-3p	6mer	no			
104	>mmu-miR-466b-3p	OS-6mer	yes			
104	>mmu-miR-466c-3p	OS-6mer	yes			
104	>mmu-miR-466m-3p	6mer	yes			

104	>mmu-miR-466o-3p	6mer	yes			
106	>mmu-miR-124-3p	7mer-m8	yes			
106	>mmu-miR-3095-3p	6mer	yes			
106				>mmu-miR-615-5p	OS-6mer	yes
107				>mmu-miR-540-5p	OS-6mer	yes
107	>mmu-miR-208a-3p	OS-6mer	yes			
107	>mmu-miR-743a-3p	OS-6mer	no			
107	>mmu-miR-743b-3p	OS-6mer	no			
107	>mmu-miR-208b-3p	OS-6mer	yes			
107	>mmu-miR-6540-5p	OS-6mer	yes			
108	>mmu-miR-7a-5p	OS-6mer	no			
108				>mmu-miR-1843b-5p	OS-6mer	yes
109	>mmu-miR-151-5p	7mer-A1	yes			
109	>mmu-miR-802-3p	7mer-A1	yes			
112	>mmu-miR-325-3p	OS-6mer	yes	>mmu-miR-325-3p	OS-6mer	yes
118	>mmu-miR-503-3p	OS-6mer	yes	>mmu-miR-503-3p	OS-6mer	yes
119	>mmu-miR-223-5p	OS-6mer	no	>mmu-miR-223-5p	OS-6mer	no
119	>mmu-miR-1198-5p	OS-6mer	yes	>mmu-miR-1198-5p	OS-6mer	yes
120	>mmu-miR-592-5p	6mer	yes	>mmu-miR-592-5p	6mer	yes
120	>mmu-miR-669a-5p	OS-6mer	yes	>mmu-miR-669a-5p	OS-6mer	yes
120	>mmu-miR-669d-5p	OS-6mer	yes	>mmu-miR-669d-5p	OS-6mer	yes
120	>mmu-miR-669f-5p	OS-6mer	yes	>mmu-miR-669f-5p	OS-6mer	yes
120	>mmu-miR-669l-5p	OS-6mer	yes	>mmu-miR-669l-5p	OS-6mer	yes
120	>mmu-miR-669p-5p	OS-6mer	yes	>mmu-miR-669p-5p	OS-6mer	yes

Supplementary Figure 5. Testing the final mutant sequence validity

A 150 nucleotide segment of the mouse *Pax6* 3'UTR was used as input into ImiRP. Six miRNA target sites predicted by PITA and TargetScan (Figure S4b) were selected for mutation. Mutation parameters were set such that three nucleotide changes would be introduced per specified target site, using all four nucleotide bases for mutation. 8mer, 7mer-m8, 7mer-A1 target sites with and without a single G:U pair and 6mer target site without a G:U pair were specified as invalid. As such, the final mutant sequence should not contain any new-created sites of these types. a) Alignment of the 150 nucleotide wild type input sequence with the mutated sequence output by ImiRP. b) Comparison of miRNA target site predictions output by ImiRP for the wild type and mutant mouse *Pax6* 3'UTR segment. All predicted miRNA target sites selected for mutation have been disrupted. Only 6mer+G:U and OS-6mer target sites were newly created upon mutation of the specified target sites.

Supplemental Document:

Instruction on How to Use ImiRP

Creating a New Project

1. Type imirp.org into your web browser. Click on the App tool at the top of the screen, and then select "Create New Project" from the Projects drop-down menu.
2. To create a new project, first provide a project name and a description of the project (optional)

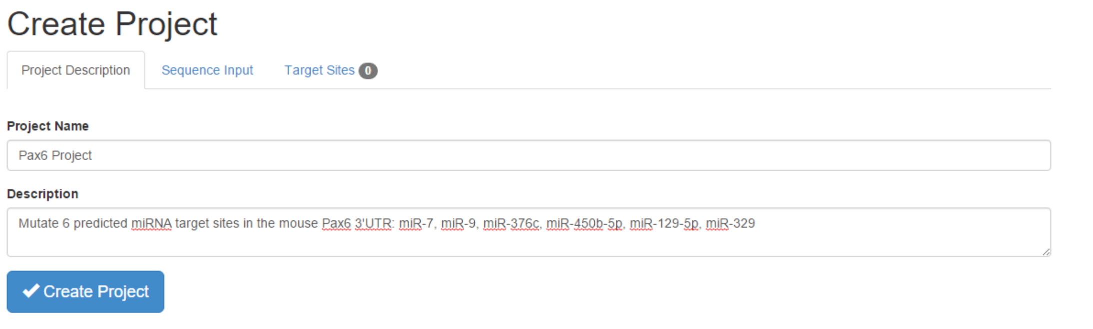

In this example, we have named the project "Pax6 Project", and will mutate six predicted target sites in a segment of the mouse *Pax6* 3'UTR.

3. Next, click on the "Sequence Input" tab of the "Create Project" menu, input the desired 3'UTR sequence and select the species of interest. A FASTA format DNA or RNA sequence can be cut and pasted into the "Sequence" text box. The input sequence is then displayed above the create project tabs. To indicate the species, begin typing the scientific name or three letter organism code into the "Species" text box. All species having miRNA sequences available in miRBase version 21 are available for selection.

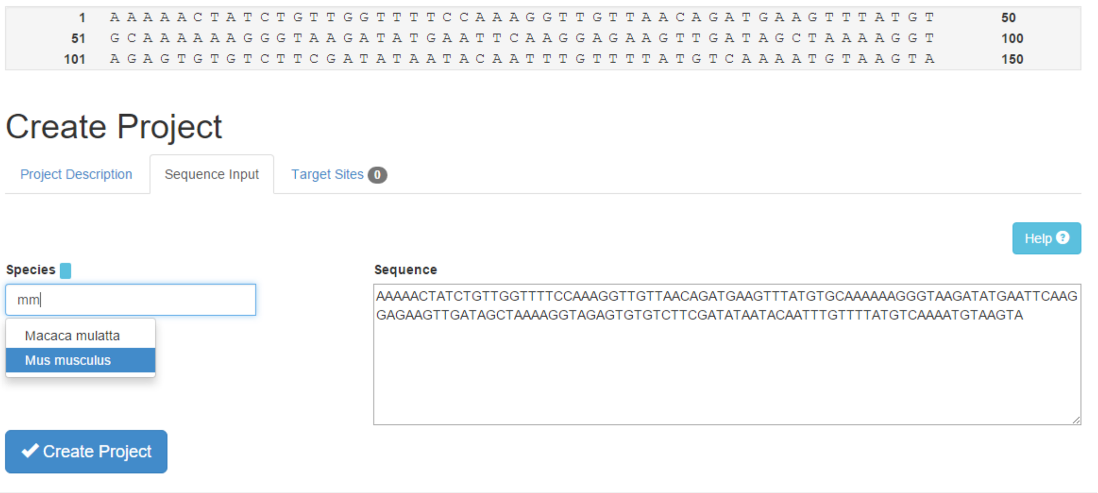

A 150 nucleotide segment of the mouse Pax6 3'UTR has been copied into the sequence text box and is displayed above the project tabs for viewing. We have begun to enter "mmu", the three letter organism code for mouse, into the species text box. Mus musculus is provided as one of the species options for selection, and can be clicked on to define mouse as the desired species.

4. Specify the regions of the input sequence, termed mutation sites, into which nucleotide substitutions will be introduced. Identify the input sequence nucleotide position of the first predicted target site to mutate that is complementary to position 7 of the targeting miRNA. Type this number into the "mutation site" text box and click "Add Site". The region of the predicted target site complementary to miRNA positions 2-7 will be bolded in the displayed input sequence. Also, the input sequence position of the start and end of the defined mutation site will be represented in (x, y) format below the mutation sites text box, where x is the first and y is the last nucleotide of the mutation site. This process is repeated for each target site to be mutated.

AAAAACTATCTGTTGGTTTTCCAAAGGTTGTTAACAGATGAAGTTTATGT
GCAAAAAAGGGTAAGATATGAATTCAAGGAGAAGTTGATAGCTAAAAGGT
AGAGTGTGTCTTCGATATAATACAATTTGTTTTATGTCAAAATGTAAGTA

Start Position	miRNA	Site Type
16	mmu-miR-7a/b	8mer+G:U
21	mmu-miR-9	6mer
44	mmu-miR-376c	7mer-m8+G:U
50	mmu-miR-450b-5p	7mer-A1
51	mmu-miR-129-5p	8mer
104	mmu-miR-329	6mer

In this example, six miRNA target sites are to be mutated. The start position indicates the first nucleotide position of each miRNA target site, with the first nucleotide of the 3'UTR being position 1. +G:U indicates that the predicted target site contains a single G:U wobble pair. For each predicted target site, the site position complementary to miRNA position 7 is typed into the mutation site text box.

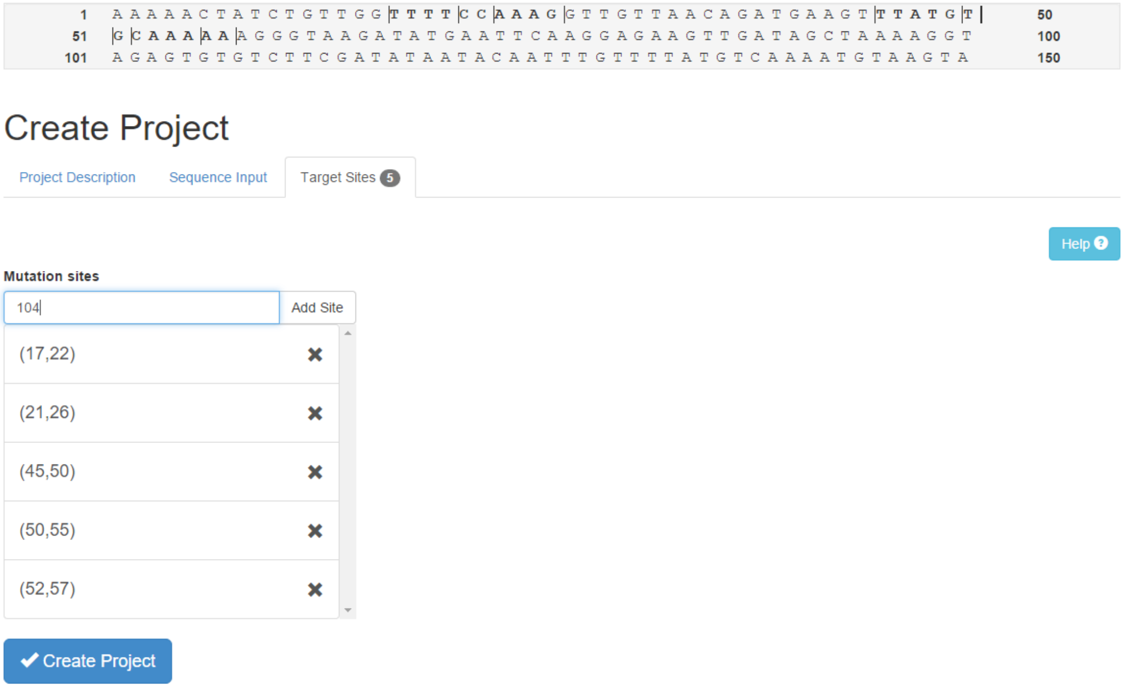

In the provided example, the mutation site for miR-7 was defined by entering 17 into the mutation site text box, and the mutation site for miR-329 is being defined by typing 104. The mutation site corresponding to each predicted target site is indicated in bold in the sequence display, and positions defining the start and end of the mutation sites are listed below the input text box. For example, the mutation site for miR-7 is listed as (17,22), and is defined in bold in the display sequence as |**TTTTCC**|. To remove a mutation site from the list, click the X.

5. Once all the project information has been defined, click the "Create Project" button at the bottom left of the page.

Defining Mutation Parameters

1. Once a project has been created, the user is directed to the project page. Here, a mutation request can be submitted.

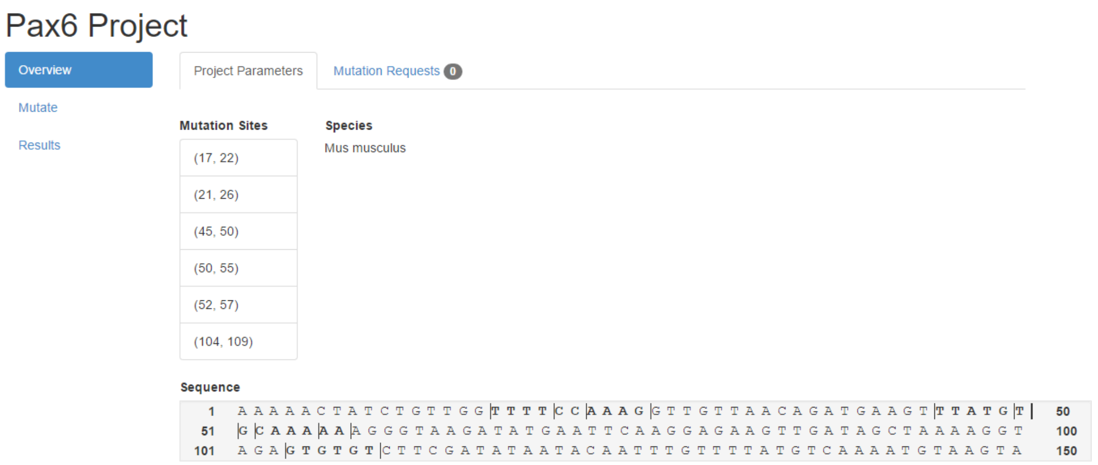

Clicking on “Overview” on the project page will provide a summary of the information that was input when the project was created. It will also provide information about any mutation requests that have been submitted. Since we have not yet submitted a mutation request the number “0” appears on the mutation request tab and no information is available for viewing.

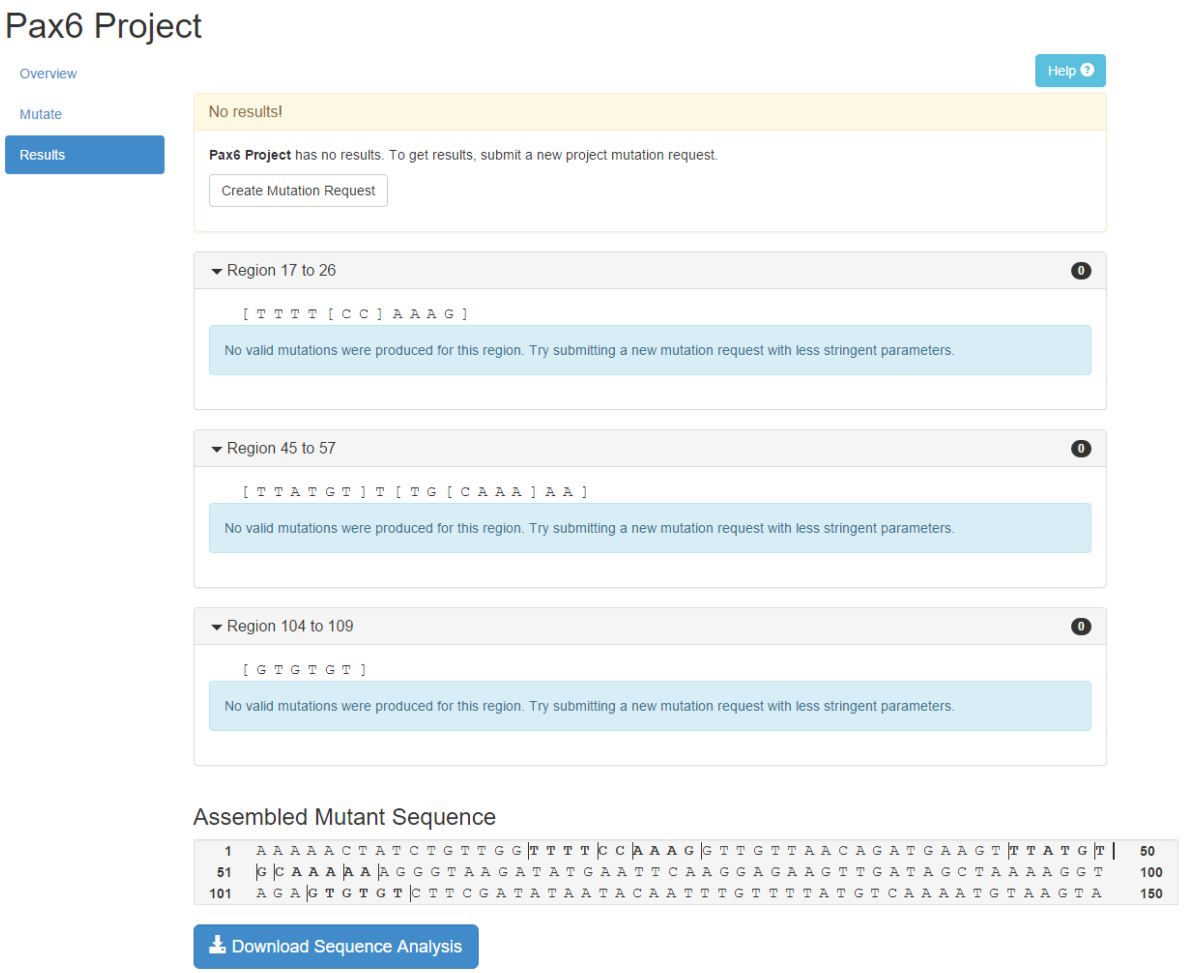

Clicking on “Results” on the project page shows that no mutant sequences have been generated because a mutation request has not been submitted. Mutation sites are grouped

based on inter-site spacing, with sites spaced less than 7 nucleotides apart being grouped into a single "Region". The nucleotide position of each region is defined based on the start and end positions of the first and last sites in the region, and the mutation sites are displayed in square brackets. For example, the mutation sites corresponding to the predicted miR-7 and miR-9 target sites are present in the first region, which begins are input sequence position 17 and ends at position 26. The mutation sites are displayed as [TTTT[CC]AAAG], with [TTTTCC] defining the mutation site for miR-7 and [CCAAAG] for miR-9.

2. Clicking the "Mutate" option on the project page enables the user to submit a mutation request.

3. Clicking on the "Mutation Strategy" tab allows the user to define the nucleotides that can be used for introducing substitutions into the mutation sites. Using the drop down menu, the user can also specify the number of nucleotide changes required per mutation site.

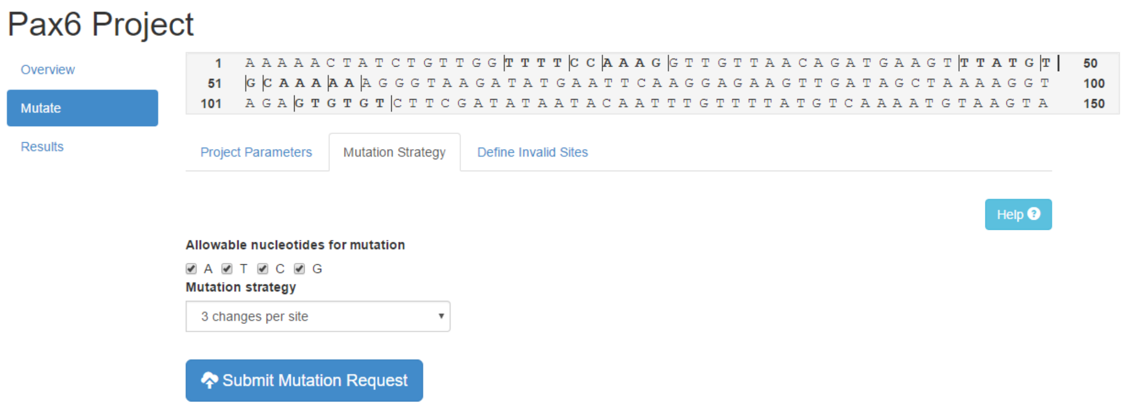

In this example, we have selected all four nucleotides for mutation and 3 changes per site. This will ensure that three adjacent nucleotide substitutions will be introduced per mutation site using any of the four available nucleotides.

4. Next, clicking on the "Define Invalid Sites" tab enables the user to specify the miRNA target site types that they want to avoid creating upon mutation of the defined mutation sites. By clicking on the target site classes listed in the "Invalid site types" and "Valid site types" lists, and using the arrow buttons, users can move target site types between the two lists.

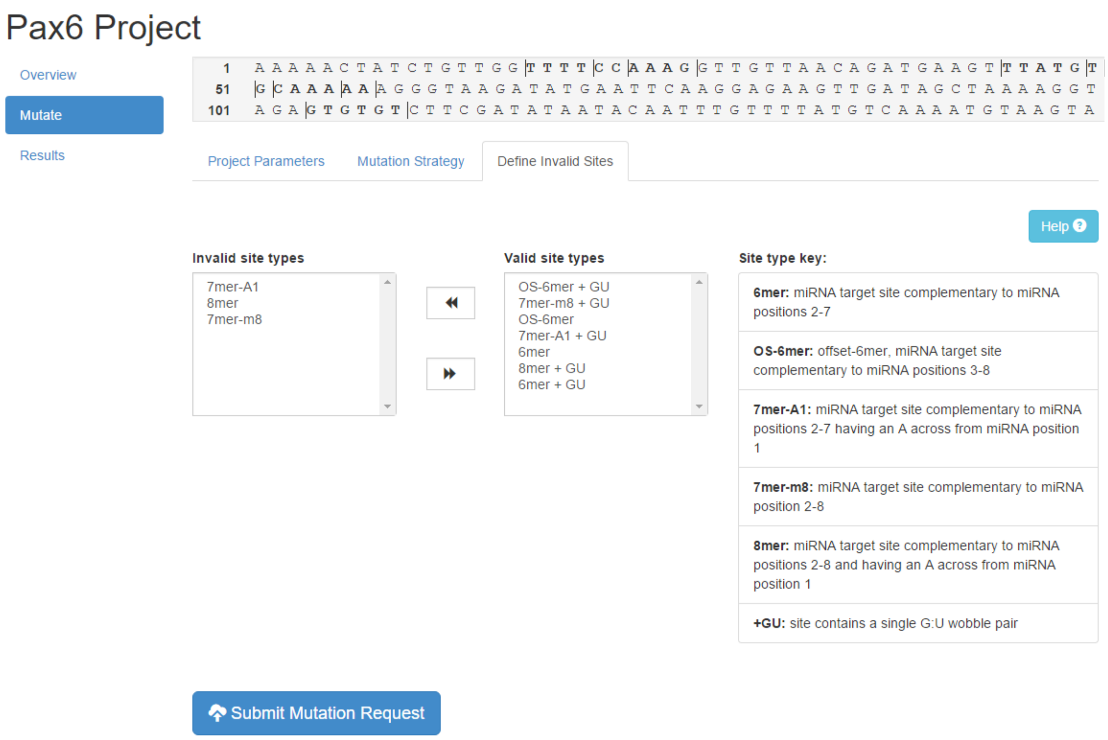

In this example, 7mer-A1, 8mer and 7mer-m8 target site types have been defined as invalid. This means that upon mutation of our six miRNA target sites, no new target sites of these types will be created. However, any target site types listed as "Valid site types" may be created upon mutation.

5. Once mutation parameters have been defined, clicking the "Submit Mutation Request" button will begin the generation of mutant sequences that satisfy the input criteria. The user will be directed to the results page within the project page.

Constructing a Final Mutant Sequence

1. A maximum of five mutations that satisfy the input mutation criteria will be displayed per region. For each region display box, the original input sequence is displayed above, and the mutated sequences are listed below.

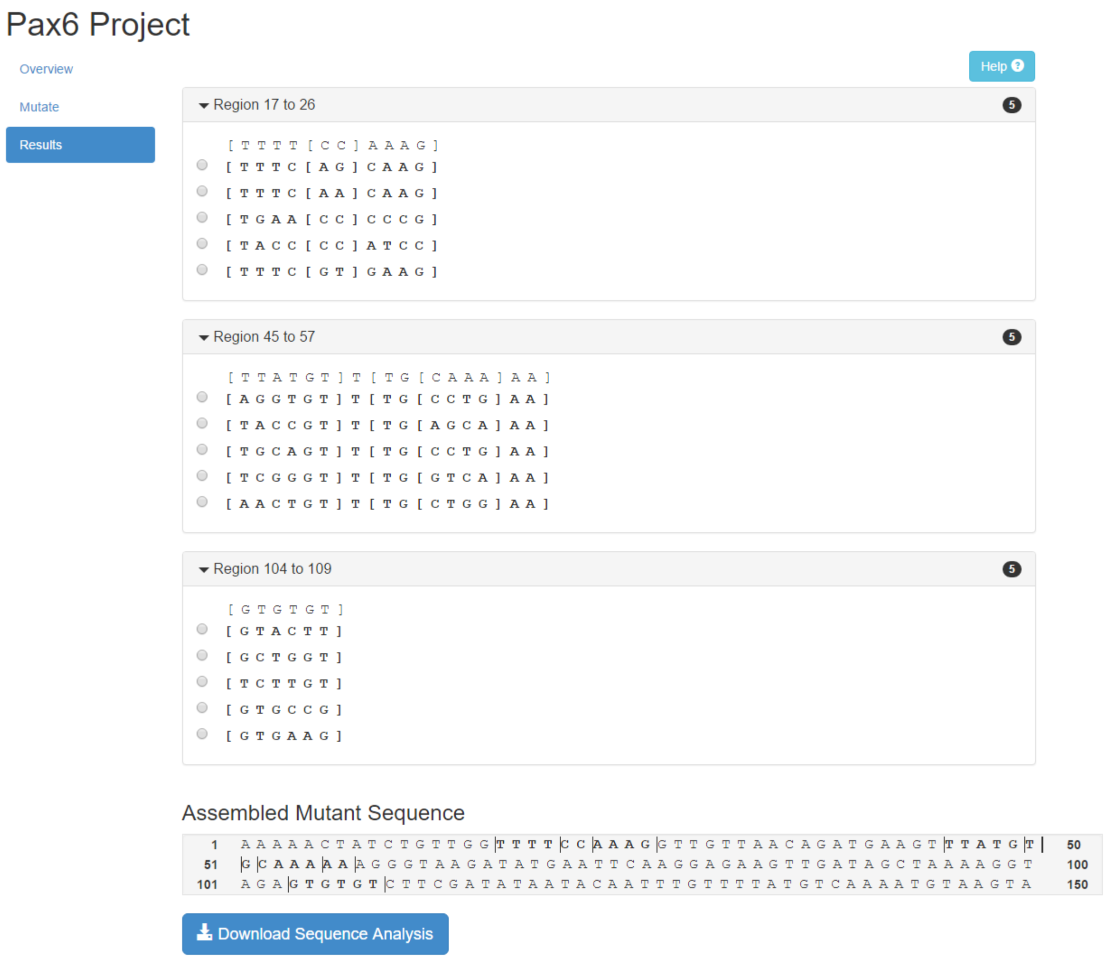

2. To select the desired mutant sequence for each region from the list, click on the select bubble to the left of the mutant sequence. One mutant sequence can be selected per region. As mutant sequences are selected for each region the region display box will change green and the “Assembled Mutant Sequence” displayed below will be updated to include the specified nucleotide substitutions.

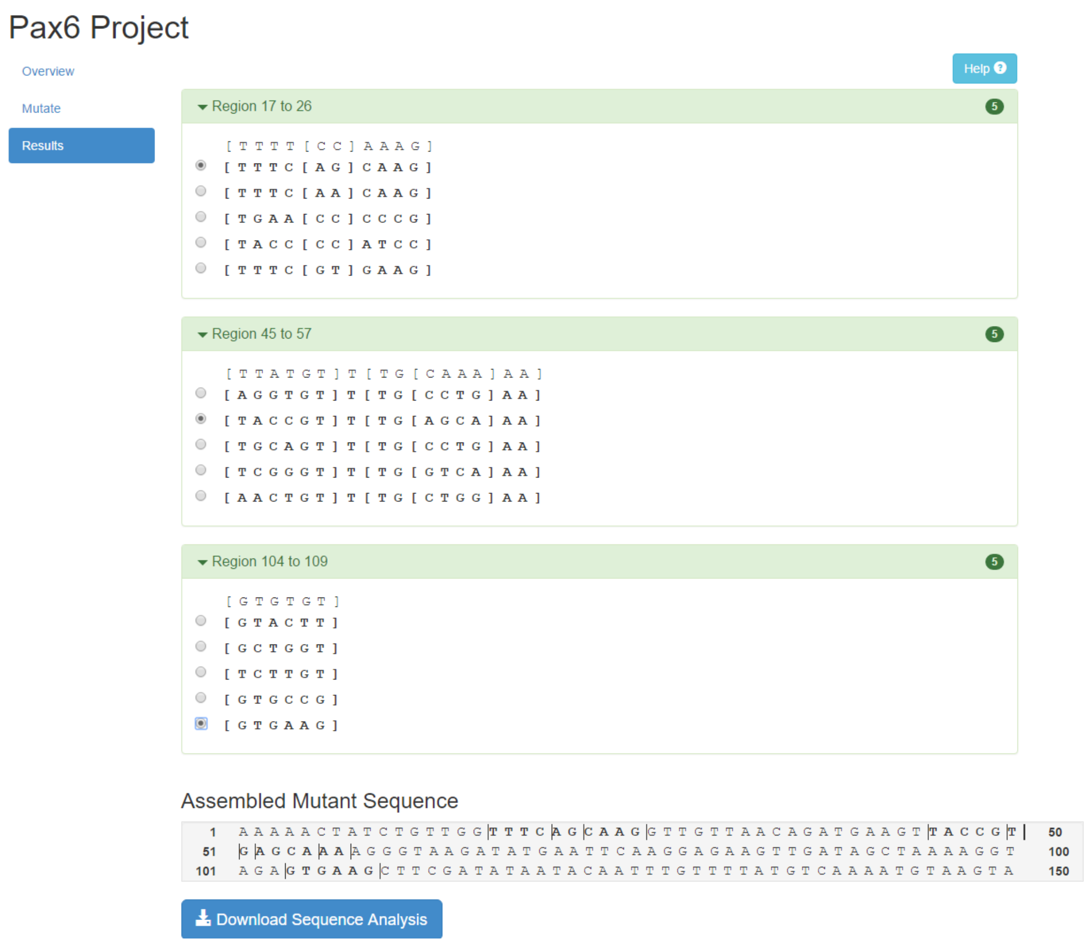

In this example, we have selected the first mutant sequence from Region 17-26 (which includes mutation sites for miR-7 and miR-9), the second mutant sequence for Region 45-57 (which includes mutation sites for miR-376c, miR-450b-5p and miR-129-5p) and the fifth mutant sequence for Region 104-109 (which includes the mutation site for miR-329). The nucleotide substitutions corresponding to these selections have now been displayed in the assembled mutant sequence.

3. Once one mutant sequence has been selected per region, clicking the "Download Sequence Analysis" button will enable the user to download a ZIP folder, entitled mutant_analysis.zip, containing a single TXT file and three CSV files. The TXT file contains the project information, input sequence and final mutant sequence. The CSV files can be opened using Excel and contain information about the predicted miRNA target sites in the input sequence, predicted target sites in the mutant sequence, and all predicted target sites that have been newly created in the mutant sequence.

If the mutation criteria are too stringent and successful mutations are not generated, returning to the mutate page within the project page will enable to user to specify new mutation
